# Locally advanced head and neck squamous cell carcinoma treatment efficacy and safety: a systematic review and network meta-analysis

**DOI:** 10.3389/fphar.2023.1269863

**Published:** 2023-09-19

**Authors:** Huanhuan Wang, Zhuangzhuang Zheng, Yangyu Zhang, Chenbin Bian, Jindian Bao, Ying Xin, Xin Jiang

**Affiliations:** ^1^ Jilin Provincial Key Laboratory of Radiation Oncology and Therapy, The First Hospital of Jilin University, Changchun, China; ^2^ NHC Key Laboratory of Radiobiology, School of Public Health, Jilin University, Changchun, China; ^3^ Division of Clinical Research, The First Hospital of Jilin University, Changchun, China; ^4^ Key Laboratory of Pathobiology, Ministry of Education, Jilin University, Changchun, China; ^5^ College of Basic Medical Sciences, Jilin University, Changchun, China

**Keywords:** locally advanced head and neck squamous cell carcinoma, meta-analysis, concurrent chemo-radiotherapy, radiotherapy, accelerated fractionated radiotherapy

## Abstract

Head and neck squamous cell carcinoma (HNSCC) accounts for approximately 3% of new cancer cases and 3% of all deaths worldwide. Most HNSCC patients are locally advanced (LA) at diagnosis. The combination of radiotherapy (RT), chemotherapy, targeted therapy, and immunotherapy are the primary LA-HNSCC treatment options. Nevertheless, the choice of optimal LA-HNSCC treatment remains controversial. We systematically searched public databases for LA-HNSCC-related studies and assess treatment effectiveness and safety by assessing the objective response rate (ORR), ≥3 adverse events (AEs), overall survival (OS), progression-free survival (PFS), disease-free survival (DFS), local-region control (LRC), and disease-specific survival (DSS). 126 randomized controlled clinical trials (RCTs) were included in this study. We show that concurrent RT with nimotuzumab or conventional concurrent chemo-radiotherapy (CCRT) had significantly better efficacy and long-term survival without increasing AEs than RT alone. Accelerated fractionated radiotherapy (AFRT) showed better efficiency than conventional fractionated RT, although it had higher AEs. In addition, concurrent cetuximab combined with RT failed to show a significant advantage over RT alone.

**Trial registration:** PROSPERO CRD42022352127.

## Highlights

Question

What is the best treatment for patients with locally advanced head and neck squamous cell carcinoma (LA-HNSCC)?

Findings

In this meta-analysis of 126 clinical trials including 22,726 patients, concurrent RT with nimotuzumab or conventional concurrent chemo-radiotherapy (CCRT) had significantly better efficacy and long-term survival without increasing AEs than RT alone. Accelerated fractionated radiotherapy (AFRT) showed better efficiency than conventional fractionated RT, although it had higher AEs. In addition, concurrent cetuximab combined with RT failed to show a significant advantage over RT alone.

Meaning

Our study provides more precise data on the treatment efficacy and safety of multiple treatment regimens among LA-HNSCC patients which facilitates clinical treatment decision making.

## Background

Head and neck cancers (HNCs) are heterogeneous tumors that include oral cavity, oropharynx, hypopharynx, larynx and several other subregions (Wyss et al., 2013; Siegel et al., 2018). Approximately 750,000 new cases and 360,000 deaths occur annually worldwide (Sung et al., 2021). Head and neck squamous cell carcinoma (HNSCC) is the most common type of HNCs. HNSCC accounts for approximately 3% of new cancer cases and 3% of deaths worldwide (Siegel et al., 2020). Approximately 30%–40% of HNSCC patients have early-stage disease (phase I/II) at diagnosis and are usually cured by surgery or radiotherapy (RT) alone (Pfister et al., 2014). More than 60% of HNSCC patients are initially diagnosed as locally advanced (LA) (Argiris et al., 2008; Braakhuis et al., 2012). The American Joint Committee on Cancer (AJCC) 8th edition staging system (2017) defines LA-HNSCC as T3-4 or N1-3 (Sahin et al., 2019). LA-HNSCC has a high risk of local recurrence and poor prognosis and is usually managed with combinations of surgery, RT, and systemic therapy (Pfister et al., 2020).

Previously, surgery combined with postoperative RT are the standard treatment options for LA-HNSCC (Kramer et al., 1987). With the progressive demand of organ function preservation, radical RT plays a crucial role in early and LA disease for maintaining swallowing and speech of LA-HNSCC patients. Compared to RT alone, concurrent RT combined with platinum-based chemotherapy significantly improves patient survival and quality of life, making it the first-line treatment option for LA-HNSCC (Sindhu and Bauman, 2019). However, platinum-based chemotherapy, particularly high-dose cisplatin, may lead to severe adverse events (AEs) in the early treatment stages. Therefore, there is an urgent need for new therapeutic options to improve HNSCC prognosis. Epidermal growth factor receptor (EGFR), a member of the tyrosine kinase receptor family, is closely associated with tumor angiogenesis, cell proliferation, invasion, metastasis, and inhibition of apoptosis (Liang et al., 2020). EGFR is highly expressed in over 90% of HNSCC (Ishitoya et al., 1989; Grandis and Tweardy, 1993) and strongly associated with poor prognosis (Rubin Grandis et al., 1998; Chung et al., 2006). Therefore, EGFR has emerged as an important therapeutic target for HNSCC (Pirker et al., 2012). Cetuximab, a chimeric murine antibody linked to human IgG and EGFR, has been confirmed to enhance the effects of RT in preclinical models (Marur and Forastiere, 2016). The IMCL-9815 study (Bonner et al., 2006) also demonstrated that combining cetuximab with RT resulted in a 13% improvement in local-region control (LRC) and a 10% improvement in overall survival (OS) compared to RT alone. Therefore, cetuximab has been approved by the Food and Drug Administration (FDA) to treat HNSCC. Nimotuzumab, a fully-human antibody against EGFR, increases clinical responses to chemo-radiotherapy (59.5% *versus* 34.2%), has a lower incidence of AEs, and has also been approved for HNSCC treatment (Rodríguez et al., 2010). In addition, induction chemotherapy (IC) is recommended for some patients, although its benefits remain controversial. In recent years, clinical trials of immunotherapy for LA-HNSCC have demonstrated the successful use of immunotherapy for recurrent/metastatic HNSCC (Seiwert et al., 2016). However, there is no conclusive evidence for application of immunotherapy in LA-HNSCC.

Currently, the combination of multiple antitumor treatment regimens targeting different mechanisms provides treatment options for a larger number of LA-HNSCC patients. Nevertheless, up to 40% of patients eventually develop distant metastases after multimodal therapy (Seiwert and Cohen, 2005; Marur and Forastiere, 2016) and have a poor prognosis (Chow, 2020). Therefore, the best treatment option for LA-HNSCC remains controversial. However, there have been no simultaneous comparisons of multiple treatment options for LA-HNSCC. We performed a systematic review and network meta-analysis to update the existing paradigm of regimen selection. This study summarized and compared multiple treatment options for LA-HNSCC to assess treatment efficacy and safety. We applied multiple indicators such as objective response rate (ORR), OS, and progression-free survival (PFS) to provide recommendations for the best treatment options suitable for LA-HNSCC patients. Notably, nasopharyngeal carcinoma, which is a unique HNSCC subtype that is usually associated with EBV infection. According to National Comprehensive Cancer Network (NCCN) guideline recommendations, the treatment options for nasopharyngeal carcinoma are significantly different compared to other HNSCCs and therefore were not included in this study.

## Methods

### Protocol and registration

The entire study process, including the original study search, completion of the systematic review, and network meta-analysis, followed the Preferred Reporting Items for Systematic Review and Meta-Analyses (PRISMA) guidelines (Supplementary Table S1) (Hutton et al., 2015). Before the start of the study, the protocol was registered with PROSPERO (CRD42022352127).

### Data sources

We systematically searched all relevant articles and references published in the PubMed, Embase, Web of Science, and Cochrane Library databases from their establishment to 24 May 2022, using the search strategy designed by experts in the epidemiology laboratory of our institution. No language restrictions were imposed. The search strategy is presented in Supplementary Table S2. To include the most comprehensive research results, we investigated randomized controlled trials (RCTs) published at recent international conferences and searched the reference lists of relevant studies and reviews for additional information.

### Eligibility criteria and data extraction

Studies that met the following criteria were included.

(1) Pathologically confirmed LA-HNSCC;
(2) No surgical treatment option;
(3) Included the outcome indicators considered in this study: ORR, ≥3 AEs, OS, PFS, disease-free survival (DFS), LRC, and disease-specific survival (DSS). ORR defined as the proportion of patients with a complete and partial response to treatment. ≥3 AEs defined as the incidence of level 3 or higher adverse events as defined by the National Cancer Institute’s Generic Term. OS defined as the time from the start of the study to the occurrence of death. PFS defined as the time from the start of the study to the patient’s first tumor progression or death. DFS defined as the time from complete clinical remission following treatment to the reappearance of focal recurrence. LRC defined as the time during which the local tumor shrank consistently or remained stable. DSS defined as the time from the start of the study to the occurrence of death by the specific disease.
(4) The study was an RCT.

In addition, we only included the most recent version that contained appropriate survival data if a study was repeatedly reported. For studies that included overlapping data from other studies, we selected a wider range of studies for analysis.

Two researchers (H. H. W. and Z. Z. Z.) independently reviewed the titles and abstracts according to the inclusion criteria to identify relevant studies for potential inclusion. Studies that potentially met the criteria were carefully reviewed to decide whether to be included in the study. Disputes were resolved by consensus with a third investigator (X. J.). According to predesigned data extraction tables discussed by the group members, two researchers (Y.Y.Z. and C.B.B.) independently extracted basic study characteristics, including study start and end times, sample size, median patient age, intervention protocol, and outcome indicators. Disagreements between the two researchers were resolved through group discussions. We directly extracted the hazard ratios (HR) and 95% confidence intervals (95% CI) of studies and calculated standard errors. For studies that failed to report HR and 95% CI, we used Digitizeit software (V4.1) to extract survival data from the survival curves and to calculate HR and standard errors. For studies with missing data, we attempted to obtain more complete information by contacting the study institution.

### Risk of bias assessment

The methodological quality of all included studies was assessed independently by two researchers (H. H. W. and Y. Y. Z.) using the Cochrane Risk Assessment Tool (Cumpston et al., 2019). This study focused on seven aspects of the original studies: whether sequence generation was random, the allocation was hidden, participants were blinded, outcome evaluators were blinded, outcome data were selectively reported, outcome data were incomplete, and other biases. Each aspect was assessed as low, high, or uncertain risk. Any disputes were discussed and resolved by a third team member.

### Statistical analysis

To evaluate the effectiveness and safety of the different treatment regimens, all evidence from direct and indirect comparisons was combined. The primary outcome indicators in this study included ORR and ≥3 AEs. The secondary outcome indicators included OS, PFS, DFS, LRC, and DSS. Odds ratios (ORs) and their 95% CIs were used to assess the effectiveness and safety of the two major outcome measures. HR and 95% CI were used as effect measures to assess the survival indicators. Considering the large number of original studies that reported primary outcome indicators, the different treatment regimens were divided into two categories: 1) neoadjuvant chemotherapy combined with concurrent chemo-radiotherapy (CCRT) and 2) systemic treatment with concurrent RT. The Bayesian method was employed to fit the model in this study because this model is more accurate and stable than the traditional classical frequency method. STATA.15 and R 3.6.0 software were applied to analyze the outcome indicators.

First, we pre-processed the data using STAT5 software and plotted a network diagram of ORRs and ≥3 AEs to visualize the direct and indirect comparisons between the different treatment regimens. The circles represent the different treatment regimens. The circle sizes are proportional to the number of patients (Chaimani et al., 2013). The line thickness indicates the number of clinical trials. Study inconsistencies were evaluated, and an effect value was calculated by separately fitting the consistency and inconsistency models. Then, different treatment regimens were ranked according to the cumulative probability ranking calculated using Bayesian analysis. Subsequently, a traditional head-to-head meta-analysis of clinical trials with the same intervention regimen was conducted, and forest plots were drawn. Study heterogeneity was evaluated using Q-tests and I^2^, with *p* < 0.05 or I^2^>50% considered significant consistency (Higgins et al., 2003). Both the fixed and random effects model was chosen based on the heterogeneity results. Finally, funnel plots were drawn to assess publication bias.

The Gemtc and Coda packages in R were applied to pre-process the data, plot survival indicator network, fit the consistency model, and create an effect value matrix (White et al., 2012). Deviance information criteria (DIC) were calculated, and Markov chain Monte Carlo (MCMC) iterations (20,000 times) were performed. Convergence diagnostic plots, trajectory plots, and distribution density plots were also drawn (Supplementary Figure S1). Heterogeneity tests were performed to assess the reliability of the results (Supplementary Figure S2). Node-splitting methods were applied to assess the inconsistency of the results and to suggest stability. Finally, the different treatment regimens were ranked according to surface under the cumulative ranking (SUCRA) values and the stacked ranking chart and individual ranking scale were plotted. All tests were bilateral. Statistical significance was set at *p* < 0.05.

## Results

### Study characteristics

**FIGURE 1:**
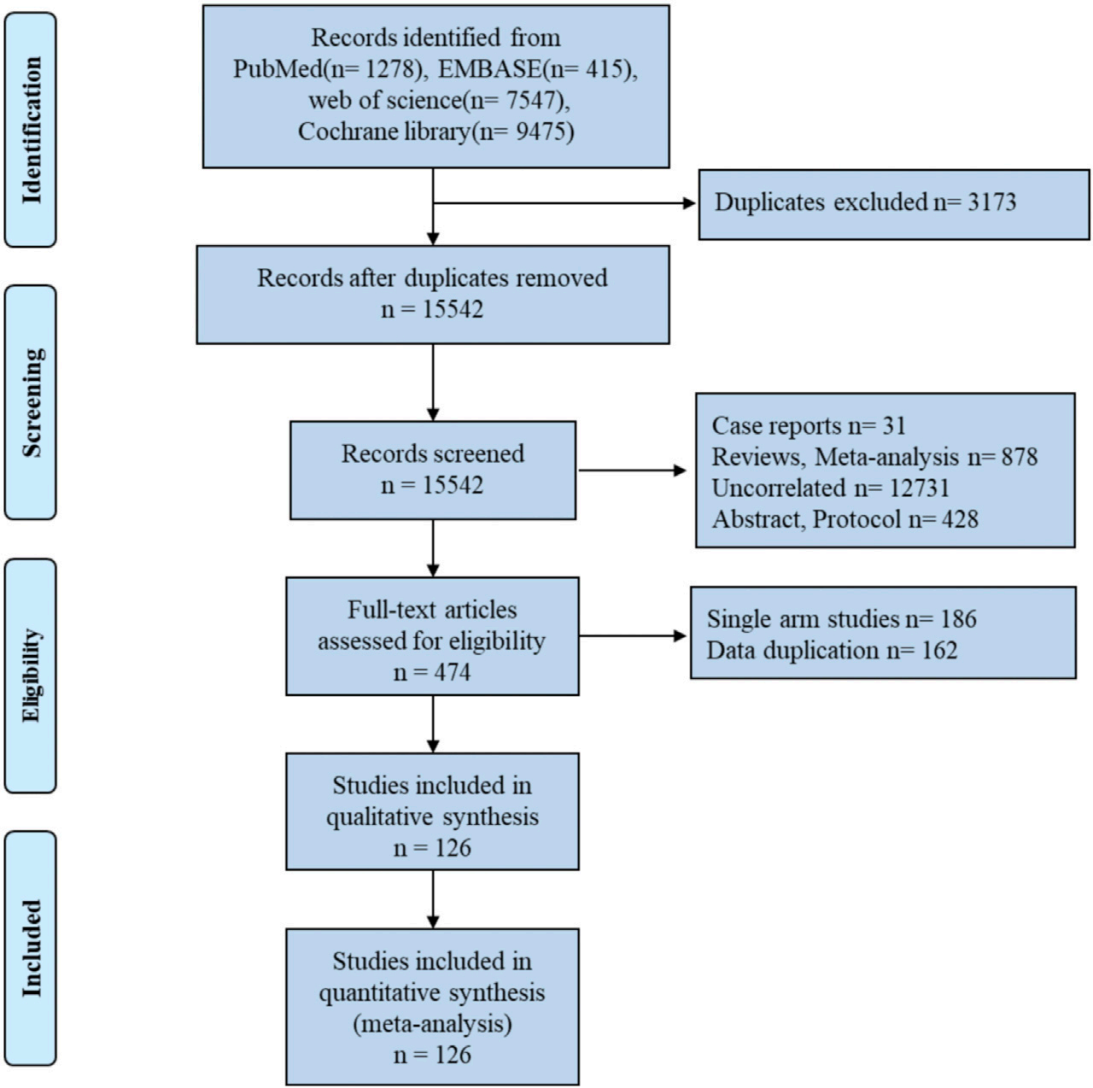
Flow diagram of article search and study selection.

We found 18,715 articles, 15,542 of which were selected through initial automatic and manual checking. A total of 15,068 articles were removed after browsing the titles and abstracts. Among these articles, 1878 were reviews and meta-analyses, 31 were case reports, 12,731 were unrelated studies, and 428 were trial design protocols. The remaining 126 RCTs were included in this study, comprising a total of 22,726 LA-HNSCC patients. The detailed process of literature search and screening is shown in Figure 1.

This study included 126 RCTs that were conducted in different countries and regions between 1975 and 2021. The basic characteristics of all the original studies included in this study are summarized in Table 1. Because no significant differences were found in the included patient populations, the transferability of this study was considered acceptable.

**TABLE 1:** Baseline characteristics included in a network meta-analysis of the treatment of locally advanced HNSCC patients.

Study	Start-stop time	Tumor site	Sample size (No)	Median age	Control arm	Intervention arm	Reported outcome
Knowlton, A. H.1975	-	HNSCC	48/48	58	RT	CCRT (Methotrexate + RT)	RR, OS, Toxicity
Percarpio, B.1976	1973.04--	HNSCC	17/18	-	RT	Altered fractionation RT	OS, DFS
Shanta, V.1977	1971--	OSCC	62/74	49.4	RT	CCRT (Bleomycin + RT)	RR, DFS
Fazekas, J. T.1980	1968–1972	HNSCC	326/312	-	RT	IC Methotrexate + (RT)	OS
Petrovich, Z.1981	1975.07–1978.02	HNSCC	11/12	59	RT	IC (Vincristine + Methotrexate) + RT	RR, OS
Weissberg, J. B. 1983	1973–1979	HNSCC	31/33	64	RT	Altered fractionation RT	RR, Toxicity
Nissenbaum, M.1984	1979.12–1981.07	HNSCC	13/22	50	RT	(Cisplatin + Bleomycin)	RR, OS
			13/23	52	RT	CCRT (Cisplatin + Bleomycin + RT)	
Bogaert, W. 1986	1981.02–1984.10	HNSCC	164/161	58	RT	Spilt Altered fractionation RT	LRC, OS
Fu, K. K.1987	1978.03–1984.08	HNSCC	51/45	57.2	RT	CCRT (Bleomycin + RT)	RR, LRC, DFS, OS, Toxicity
Klima, A.1988	-	HNSCC	44/36	52	(Cisplatin + Bleomycin + Methotrexate)	IC (Cisplatin + Bleomycin + Methotrexate) + RT	RR, OS
Sanchiz, F.1990	1978.01–1988.01	HNSCC	294/292	56	RT	Altered fractionation RT	RR, PFS, OS, Toxicity
			294/306	-	RT	CCRT (5-FU + RT)	
Pinto, L. H.1991	1986.04–1989.05	OPSCC	48/50	56	RT	Altered fractionation RT	RR, OS, DFS
Tejedor, M.1992	1987.01–1989.07	HNSCC	17/19	57.4	RT	IC (Carboplatin + Ftorafur) + RT	RR, OS, DFS, Toxicity
Weissler, M. C.1992	-	HNSCC	15/17	52	Spilt Altered fractionation RT	Spilt CCRT (Cisplatin + 5-FU + RT)	RR, OS, PFS, DSS, Toxicity
Browman, G. P.1994	1987.04–1991.08	HNSCC	87/88	61	RT	CCRT (5-FU + RT)	RR, OS, PFS, Toxicity
Kamioner, D.1994	1990.09–1992.09	HNSCC	17/20	55	CCRT (Cisplatin + RT)	CCRT (Carboplatin + RT)	RR
Pinnarò, P.1994	1986.02–1991.02	HNSCC	49/44	57	CCRT (Cisplatin + RT)	IC (Cisplatin + 5-FU) + RT	PFS, OS, LRC, RR, Toxicity
Smid, L.1995	1991.03–1993.10	HNSCC	25/24	50	RT	CCRT (Mitomycin C + Bleomycin + RT)	RR, DFS, Toxicity
Antognoni, P.1996	1992.09–1993.09	OPSCC, OSCC	46/23	60	RT	Altered fractionation RT	OS, LRC, Toxicity
Haddad, E.1996	1987.04–1992.10	HNSCC	28/28	-	IC (Cisplatin + 5-FU) + RT	IC (Cisplatin + 5-FU) + CCRT (Cisplatin + 5-FU + RT)	RR
Merlano, M.1996	1987.02–1990.12	HNSCC	77/80	-	RT	Alternate CCRT (Cisplatin + 5-FU + RT)	RR, LRC, PFS, OS, Toxicity
Horiot, J. C.1997	1985.12–1995.04	HNSCC	253/247	57	RT	Altered fractionation RT	RR, LRC, OS, Toxicity
Jackson, S. M.1997	1991.10–1995.04	HNSCC	40/40	61	RT	Altered fractionation RT	RFS, DSS, RR, Toxicity
Jeremic, B.1997	1988.01–1990.12	HNSCC	53/53	59	RT	CCRT (Cisplatin + RT)	RR, OS, LRC, Toxicity
			53/53		RT	CCRT (Carboplatin + RT)	
Benasso, M.1997	1983.08–1990.12	HNSCC	77/80	-	RT	Alternate CCRT (Cisplatin + 5-FU + RT)	RR, OS
			55/61		IC (Vinblastine + Bleomycin + Methotrexate) + RT	Alternate CCRT (Vinblastine + Bleomycin + Methotrexate + RT)	
Zakotnik, B. 1998	1991.03–1993.12	HNSCC	32/32	51	RT	CCRT (Mitomycin C + Bleomycin + RT)	DFS, OS, RR, Toxicity
Wendt, T. G.1998	1989.11–1993.10	HNSCC	140/130	51	Spilt Altered fractionation RT	Spilt CCRT (Cisplatin + 5-FU + RT)	OS, LRC, Toxicity
Calais, G.1999	1994.07–1997.09	OPSCC	113/109	56	RT	CCRT (Carboplatin + 5-FU + RT)	OS, DFS, Toxicity
Santarelli, M.1999	1992–1997	HNSCC	66/61		CCRT (Carboplatin + Etoposide + RT)	CCRT (5-FU + Mitomycin C + RT)	OS, Toxicity
Dobrowsky, W. 2000	1990.10–1997.12	HNSCC	81/78	56	RT	Altered fractionation RT	RR, OS, Toxicity
			81/80	56	RT	CCRT (Mitomycin C + Altered fractionation RT)	
Jeremic, B. 2000	1991.01–1993.03	HNSCC	65/65	61	Altered fractionation RT	CCRT (Cisplatin + Altered fractionation RT)	OS, PFS, LRC, Toxicity
Poulsen, M. G.2001	1991–1998	HNSCC	171/172	62	RT	Altered fractionation RT	DFS, LRC, RR, DSS
Bartelink, H. 2002	-	HNSCC	24/25	-	CCRT (Cisplatin + RT)	Spilt CCRT (cisplatin + Altered fractionation RT)	OS, LRC, Toxicity
Adelstein, D. J.2003	1992.03–1999.12	HNSCC	95/87	57	RT	CCRT (Cisplatin + RT)	RR, OS, DSS, Toxicity
Grau, C.2003	1996.02–1999.12	HNSCC	221/245	55	RT	CCRT (Mitomycin C + RT)	DSS, LRC, Toxicity
Denis, F.2004	-	OPSCC	112/108	55	RT	CCRT (Carboplatin + 5-FU + RT)	OS, DFS, LRC, Toxicity
Garden, A. S.2004	1997.07–1999.06	HNSCC	78/77	56	CCRT (Cisplatin + 5-FU + RT)	CCRT (Paclitaxel + Cisplain + RT)	RR, DFS, OS, Toxicity
Fountzilas, G.2004	1995.01–1999.07	HNSCC	41/45	57	RT	CCRT (Cisplatin + RT)	PFS, OS, RR
			41/38	56	RT	CCRT (Carboplatin + RT)	
Ezzat, M.2005	1998.05–2001.10	HNSCC	20/20	53.5	RT	Altered fractionation RT	RR, OS, LRC, Toxicity
			20/20	51	RT	CCRT (Mitomycin C + Altered fractionation RT)	
Bensadoun, R. J. 2006	1997.11–2002.03	OPSCC, HPSCC	82/81	54	Altered fractionation RT	CCRT (Cisplain + 5-FU + Altered fractionation RT)	RR, OS, DFS, DSS, Toxicity
Bourhis, J. 2006	1994.11–1998.09	HNSCC	129/137		RT	Altered fractionation RT	OS, LRC, Toxicity
Fallai, C.2006	1993.01–1998.06	OPSCC	63/64	56.1	RT	CCRT (Carboplatin + 5-FU + RT)	OS, DFS, Toxicity
Mitra, D.2006	1998.08–1999.07	HNSCC	90/90	56	RT	IC (Cisplatin + 5-FU) + RT	RR, OS, Toxicity
Semrau, R. 2006	1995.07–1999.04	HNSCC	127/113	57	Altered fractionation RT	CCRT (Carboplatin + 5-FU + Altered fractionation RT)	RR, LRC, OS, Toxicity
Turcato, G.2006	-	HNSCC	42/42	-	CCRT (Cisplatin + 5-FU + RT)	IC (Docetaxel + Cisplatin + 5-FU) + CCRT (Cisplatin + 5-FU + RT)	RR, Toxicity
Manocha, S.2006	1998–2000	HNSCC	25/25	53	RT	Alternate CCRT (Cisplatin + 5-FU + RT)	RR
Katori, H.2007	2000.01–2004.04	HNSCC	25/25	58	CCRT (Docetaxel + Cisplatin + 5-FU + RT)	CCRT (Docetaxel + Cisplatin + 5-FU + Altered fractionation RT)	RR, DFS, LRC, OS, Toxicity
Chauhan, A.2008	2000.11–2003.03	HNSCC	40/40	-	RT	CCRT (Gemcitabine + RT)	RR, Toxicity
Sarkar, S. K.2008	2005.01–2006.01	HNSCC	40/32	-	CCRT (Cisplatin + RT)	CCRT (Vinorelbine + RT)	RR, Toxicity
Gupta, D.2009	2005.03–2007.07	OPSCC	57/48	50	CCRT (Cisplatin + RT)	IC (Cisplatin + 5-FU) + CCRT (Cisplatin + RT)	RR, OS, DFS, Toxicity
Bonner, J. A.2010	1999.04–2002.03	HNSCC	213/211	57	RT	Cetuximab + RT	OS, Toxicity
Devleena, D. M.2010	2004.09–2005.07	HNSCC	20/20	60	CCRT (Cisplatin + RT)	CCRT (Vinorelbine + RT)	RR
Osorio R.M. 2010	2002.07–2007.02	HNSCC	51/54	63	RT	CCRT (Nimotuzumab + RT)	OS, RR
Paccagnella, A. 2010	2003.01–2006.01	HNSCC	51/50	59	CCRT (Cisplatin + 5-FU + RT)	IC (Docetaxel + Cisplatin + 5-FU) + CCRT (Cisplatin + 5-FU + RT)	RR, PFS, OS, Toxicity
Redda, M.G.R.2010	1992.11–1995.12	HNSCC	77/80	60	RT	CCRT (Carboplatin + RT)	RR, DFS, OS, LRC, Toxicity
Campo, J. M. 2011	2006.03–2007.07	HNSCC	36/71	57	CCRT (Cisplatin + RT)	IC Lapatinib + CCRT (Cisplatin + RT)	RR, Toxicity
Bourhis, J.2011	1996–2000	HNSCC	56/53	52	Altered fractionation RT	Spilt CCRT (Cisplatin + 5-FU + Altered fractionation RT)	OS, Toxicity
Ghali, R. R.2011	2007.08–2009.09	HNSCC	30/30	-	CCRT (Cisplatin + RT)	IC (Docetaxel + Cisplatin + 5-FU) + CCRT (Cisplatin + RT)	RR, Toxicity
Gregoire, V. 2011	2004.11–2008.06	HNSCC	116/110	53.2	CCRT (Cisplatin + RT)	CCRT (Cisplatin + Gefitinib + RT)	OS, PFS, RR, LRC, Toxicity
Hamed, R. H.2011	2009.01–2010.06	HNSCC	26/25	58	CCRT (Cisplatin + RT)	CCRT (Paclitaxel + RT)	RR, LRC, PFS, OS, Toxicity
Satapathy, B.2011	2007.09–2009.11	HNSCC	25/25	55	CCRT (Cisplatin + RT)	IC (Cisplatin + 5-FU) + CCRT (Cisplatin + RT)	RR
Lorch, J. H. 2011	1999.05–2003.12	HNSCC	246/255	55	IC (Cisplatin + 5-FU) + CCRT (Carboplatin + RT)	IC(Docetaxel + Cisplatin + 5-FU) + CCRT (Carboplatin + RT)	RR, OS, PFS, Toxicity
Kumar, S.2011	1995.08–1999.03	HNSCC	95/92	56	RT	IC Cisplatin + (RT)	RR, Toxicity
			95/95		RT	CCRT (Cisplatin + RT)	
Bourhis, J.2012	2000.02–2007.05	HNSCC	279/280	56.5	CCRT (Carboplatin + 5-FU + RT)	CCRT (Carboplatin + 5-FU + Altered fractionation RT)	PFS, OS, LRC, Toxicity
			279/281	56.3	CCRT (Carboplatin + 5-FU + RT)	Altered fractionation RT	
Halim, A. F.2012	2004.03–2006.12	HNSCC	110/106	51	CCRT (Gemcitabin + RT)	CCRT (Paclitaxel + RT)	RR, PFS, OS, Toxicity
Choudhury, K.2012	2008.05–2012.05	HNSCC	46/42	-	RT	Altered fractionation RT	RR, DFS, OS, Toxicity
Chitapanarux, I.2013	2003.01–2007.12	HNSCC	48/37	61	CCRT (Carboplatin + 5-FU + RT)	Altered fractionation RT	OS, LRC, Toxicity
Gupta, S.2013	2004–2005	HNSCC	67/71	52	IC (Paclitaxel + Cisplatin) + CCRT (Cisplatin + 5-FU + RT)	IC (Paclitaxel + Cisplatin) + CCRT (Cisplatin + Capecitabine + RT)	RR, DFS, PFS, OS, Toxicity
Haddad, R.2013	2004.08–2008.12	HNSCC	75/70	54	CCRT (Cisplatin + RT)	IC (Docetaxel + Cisplatin + 5-FU) + CCRT (Docetaxel/Carboplatin + RT)	PFS, OS, Toxicity
Harrington, Kevin.2013	2006.11–2009.01	HNSCC	33/34	56	CCRT (Cisplatin + RT)	CCRT (Cisplatin + Lapatinib + RT)	PFS, OS, LRC, RR, Toxicity
Martins, R. G.2013	2006.12–2011.10	HNSCC	105/99		CCRT (Cisplatin + RT)	CCRT (Erlotinib + Cisplatin + RT)	RR, PFS, Toxicity
Rishi, A.2013	2006.07–2010.06	OPSCC	106/110	51	CCRT (Cisplatin + RT)	Altered fractionation RT	RR, DFS, Toxicity
Singh, P. K.2013	2008.12–2010.08	OSCC	30/30	55	RT	CCRT (Geftinib + RT)	RR, DFS, Toxicity
Bhattacharya, B.2014	2011.01–2012.06	HNSCC	31/30	-	CCRT (Cisplatin + RT)	CCRT (Gefitinib + Cisplatin + RT)	RR, Toxicity
Hitt, R.2014	2002.12–2007.05	HNSCC	128/155	57	CCRT (Cisplatin + RT)	IC (Docetaxel + Cisplatin + 5-FU) + CCRT (Cisplatin + RT)	RR, PFS, OS, Toxicity
			128/156	57	CCRT (Cisplatin + RT)	IC (Cisplatin + 5-FU) + CCRT (Cisplatin + RT)	
Reddy, B. K. 2014	2004.09–2005.07	HNSCC	20/20	51.8	CCRT (Cisplatin + RT)	CCRT (Cisplatin + Nimotuzumab + RT)	RR, OS, Toxicity
			19/17	58.7	RT	CCRT (Nimotuzumab + RT)	
Singh, M.2014	-	OPSCC	25/25	-	CCRT (Cisplatin + RT)	CCRT (Cisplatin + 5-FU + RT)	RR, Toxicity
Ang, K. K.2014	2005.11–2009.03	HNSCC	447/444	57	CCRT (Cisplatin + Altered fractionation RT)	CCRT (Cisplatin + Cetuximab + Altered fractionation RT)	OS, PFS, Toxicity
Nguyen. T. P. F. 2014	2002.02–2005.06	HNSCC	361/360	56	CCRT (Cisplatin + RT)	CCRT (Cisplatin + Altered fractionation RT)	OS, PFS, Toxicity
Miszczyk, L.2014	2003.03–2009.09	HNSCC	37/39	57	RT	Spilt Altered fractionation RT	RR, OS
Budach, V.2015	1995.03–1999.06	HNSCC	194/190	55	Altered fractionation RT	CCRT (Mitomycin C + 5-FU + Altered fractionation RT)	OS, LRC, DSS
Chhatui, B. 2015	2008.09–2010.12	OSCC	25/25	50	CCRT (Cisplatin + RT)	IC (Cisplatin + 5-FU) + CCRT (Cisplatin + RT)	RR, DFS, OS, Toxicity
Kumar, A. 2015	2012.03–2013.10	HNSCC	55/56	54	Altered fractionation RT	CCRT (Cisplatin + Altered fractionation RT)	RR, PFS, OS, Toxicity
Lee, K. W. 2015	-	HNSCC	44/48	-	IC (Docetaxel + Cisplatin) + CCRT (Cisplatin + RT)	IC (Docetaxel + Cetuximab + Cisplatin) + CCRT (Cetuximab + Cisplatin + RT)	RR, OS, PFS
Mesía, R.2015	2007.10–2009.03	HNSCC	63/87	-	CCRT (Cisplatin + RT)	CCRT (Panitumumab + Cisplatin + RT)	RR, LRC, PFS, OS, Toxicity
Takacsi. N. Z.2015	2007.01–2009.06	HNSCC	33/33	56	CCRT (Cisplatin + RT)	IC (Docetaxel + Cisplatin + 5-FU) + CCRT (Cisplatin + RT)	RR, OS, PFS, Toxicity
Rodriguez, C. P. 2015	2008.02–2011.10	HNSCC	35/34	57	CCRT (Cisplatin + RT)	CCRT (Cisplatin + 5-FU + RT)	OS, RFS
Gupta, M.2015	2011.04–2013.05	HNSCC	67/66	57	CCRT (Cisplatin + RT)	Altered fractionation RT	DFS, OS, RR, Toxicity
Giralt, J.2015	2007.11–2009.11	HNSCC	61/90	-	CCRT (Cisplatin + Altered fractionation RT)	CCRT (Panitumumab + Cisplatin + Altered fractionation RT)	LRC, PFS, OS, Toxicity
Argiris, A.2016	2012.01–2014.09	HNSCC	37/41	56	CCRT (Cetuximab + Pemetrexed + RT)	CCRT (Cetuximab + Pemetrexed + Bevacizumab + RT)	PFS, OS, Toxicity
Das, R.2016	-	HNSCC	29/28	-	CCRT (Cisplatin + RT)	CCRT (Cisplatin + Altered fractionation RT)	RR, Toxicity
Driessen, C. M. L. 2016	2008.12–2012.02	HNSCC	27/29	53.4	IC (Docetaxel + Cisplatin + 5-FU) + CCRT (Cisplatin + RT)	IC (Docetaxel + Cisplatin + 5-FU) + CCRT (Cisplatin + Altered fractionation RT)	PFS, OS, RR, LRC, Toxicity
Ghosh-Laskar, S.2016	2000.04–2007.10	HNSCC	57/65	56	RT	CCRT (Cisplatin + RT)	LRC, DFS, OS, Toxicity
Hitt, R.2016	-	HNSCC	205/202	56	IC (Docetaxel + Cisplatin + 5-FU) + CCRT (Cisplatin + RT)	IC (Docetaxel + Cisplatin + 5-FU) + (Cetuximab + RT)	RR, Toxicity
Magrini, S. M.2016	2011.01–2014.08	HNSCC	35/35	64	CCRT (Cisplatin + RT)	(Cetuximab + RT)	LRC, OS, DSS, Toxicity
Mishra, H.2016	2012.02–2013.02	HNSCC	15/15	53	CCRT (Paclitaxel + Cisplatin + RT)	Altered fractionation RT	RR, Toxicity
Seiwert, T. Y. 2016	2006.09–2010.04	HNSCC	57/53	57	IC (Carboplatin + Paclitaxel + Cetuximab) + CCRT (Hydroxyurea + 5-FU + Cetuximab + Altered fractionation RT)	IC (Carboplatin + Paclitaxel + Cetuximab) + CCRT (Cisplatin + Cetuximab + Altered fractionation RT)	OS, PFS, Toxicity
Tao, Y.2016	2010.01–2012.01	LSCC	25/25	55	IC (Pemetrexed + Cisplatin) + RT	IC (5-FU + Cisplatin) + RT	RR, OS, Toxicity
Ghi, M. G.2017	2008.03–2012.04	HNSCC	206/208	60.5	CCRT (Cisplatin + 5-FU + RT)/(Cetuximab + RT)	IC (Cocetaxel + Cisplatin + 5-FU) + CCRT (Cisplatin + 5-FU + RT)/(Cetuximab + RT)	RR, OS, PFS, Toxicity
He, X.2017	-	HNSCC	56/52		CCRT (Cisplatin + 5-FU + RT)	CCRT (Raltitrexed + Cisplatin + RT)	RR
Specenier, P. M.2017	2008.04–2009.12	HNSCC	15/15	56.7	IC (Docetaxel + Cisplatin + 5-FU + Cetuximab) + CCRT (Cetuximab + Cisplatin + RT)	IC (Docetaxel + Cisplatin + 5-FU + Cetuximab) + CCRT (Cetuximab + Carboplatin + RT)	RR, Toxicity
Zhe. T. M.2017	2013.06–2014.03	HPSCC	44/39	56	RT	CCRT (Paclitaxel + RT)	RR, Toxicity
Geoffrois, L. 2018	2009.05–2013.08	HNSCC	179/151	56	CCRT (Carboplatin + 5-FU + RT)	IC (Docetaxel + Cisplatin + 5-FU) + (Cetuximab + RT)	PFS, OS, LRC, Toxicity
Saini, S. K.2018	-	HNSCC	32/35	52	CCRT (Cisplatin + RT)	CCRT (Cisplatin + Gefitinib + RT)	RR, OS, PFS, Toxicity
Sun, X. S.2018	GORTEC 2015–01	HNSCC	66/67	65	(Cetuximab + RT)	CCRT (Pembrolizumab + RT)	Toxicity
Tao, Y.2018	2008.01–2014.03	HNSCC	202/204	57	(Cetuximab + RT)	CCRT (Carboplatin + 5-FU + cetuximab + RT)	OS, PFS, Toxicity
Al-Saleh, K.2019	2012.11–2017.11	HNSCC	22/18	51	CCRT (Cisplatin + Altered fractionation RT)	CCRT (Cetuximab + Altered fractionation RT)	RR, DFS, OS, Toxicity
Gillison, M. L.2019	2011.06–2014.07	OPSCC	406/399	57.6	CCRT (Cisplatin + Altered fractionation RT)	CCRT (Cetuximab + Altered fractionation RT)	OS, PFS, LRC, Toxicity
Keil, F.2019	-	HNSCC	49/51	-	IC (Docetaxel + Cisplatin + 5-FU) + (Cetuximab + RT)	IC (Docetaxel + Cisplatin + Cetuximab) + (Cetuximab + RT)	RR
Patil, V. M. 2019	2012–2018	HNSCC	268/268	54.5	CCRT (Cisplatin + RT)	CCRT (Cisplatin + Nimotuzumab + RT)	OS, PFS, DFS, LRC, Toxicity
Rastogi, M.2019	2006.03–2008.03	HNSCC	161/161	52	CCRT (Cisplatin + RT)	Altered fractionation RT	LRC, DFS, OS, Toxicity
Yom, S. S.2019	2014.10–2017.02	OPSCC	157/149		CCRT (Cisplatin + RT)	Altered fractionation RT	Toxicity
Tao, Y.2020	2017.09–2018.08	HNSCC	21/21	61.4	CCRT (Cisplatin + RT)	CCRT (Avelumabe + Cetuximabe + RT)	Toxicity
			20/20	61.4	CCRT (Avelumabe + Cetuximabe + RT)	(Cetuximabe + RT)	
Fietkau, R.2020	2010–2015.02	HNSCC	111/105	59	CCRT (Paclitaxel + Cisplatin + RT)	CCRT (Cisplatin + 5-FU + RT)	DFS, OS, Toxicity
Jones, D. A.2020	2012.11–2016.11	OPSCC	166/168	57.5	CCRT (Cisplatin + RT)	(Cetuximab + RT)	OS
Lim, S. H.2020	2010.12–2015.04	OPSCC, HPSCC	19/17	61	CCRT (Cisplatin + RT)	IC (Docetaxel + Cisplatin + 5-FU) + CCRT (Cisplatin + RT)	PFS, OS, RR, Toxicity
Maddalo, M.2020	2011.01–2014.08	HNSCC	35/35	64	CCRT (Cisplatin + RT)	(Cetuximab + RT)	OS, LRC, DSS, Toxicity
Merlano, M. C. 2020	2009.09–2016.12	HNSCC	121/121	59.5	CCRT (Cisplatin + RT)	IC (Docetaxel + Cisplatin + 5-FU) + (Cetuximab + RT)	OS, PFS, RR, Toxicity
Bourhis, J.2020	2016.05–2017.10	HNSCC	65/66	65	(Cetuximab + RT)	CCRT (Pembrolizumab + RT)	PFS, OS, Toxicity
Cohen, E. E.2020	2016.12–2019.01	HNSCC	347/350	-	CCRT (Cisplatin + RT)	CCRT (Avelumab + Cisplatin + RT)	PFS, Toxicity
Durga H. K. 2020	-	HNSCC	26/24	-	CCRT (Cisplatin + RT)	CCRT (Paclitaxel + RT)	RR
Gupta, P.2020	-	HNSCC	30/30	58.1	CCRT (Cisplatin + RT)	CCRT (Cisplatin + Altered fractionation RT)	RR, DFS, Toxicity
			30/30		CCRT (Cisplatin + RT)	Altered fractionation RT	
Gebre-M. M.2021	2013.11–2018.03	HNSCC	145/146	61	CCRT (Cisplatin + RT)	(Cetuximab + RT)	OS, Toxicity

As expected, the methodological quality assessment identified that most of the studies were low risk in terms of random sequence generation and selective reporting. Most studies did not clarify whether blinding and allocation concealment were used. Therefore, we believe that selection bias may exist. The detailed bias risk assessment results are shown in Supplementary Figure S3.

### ORR

**FIGURE 2:**
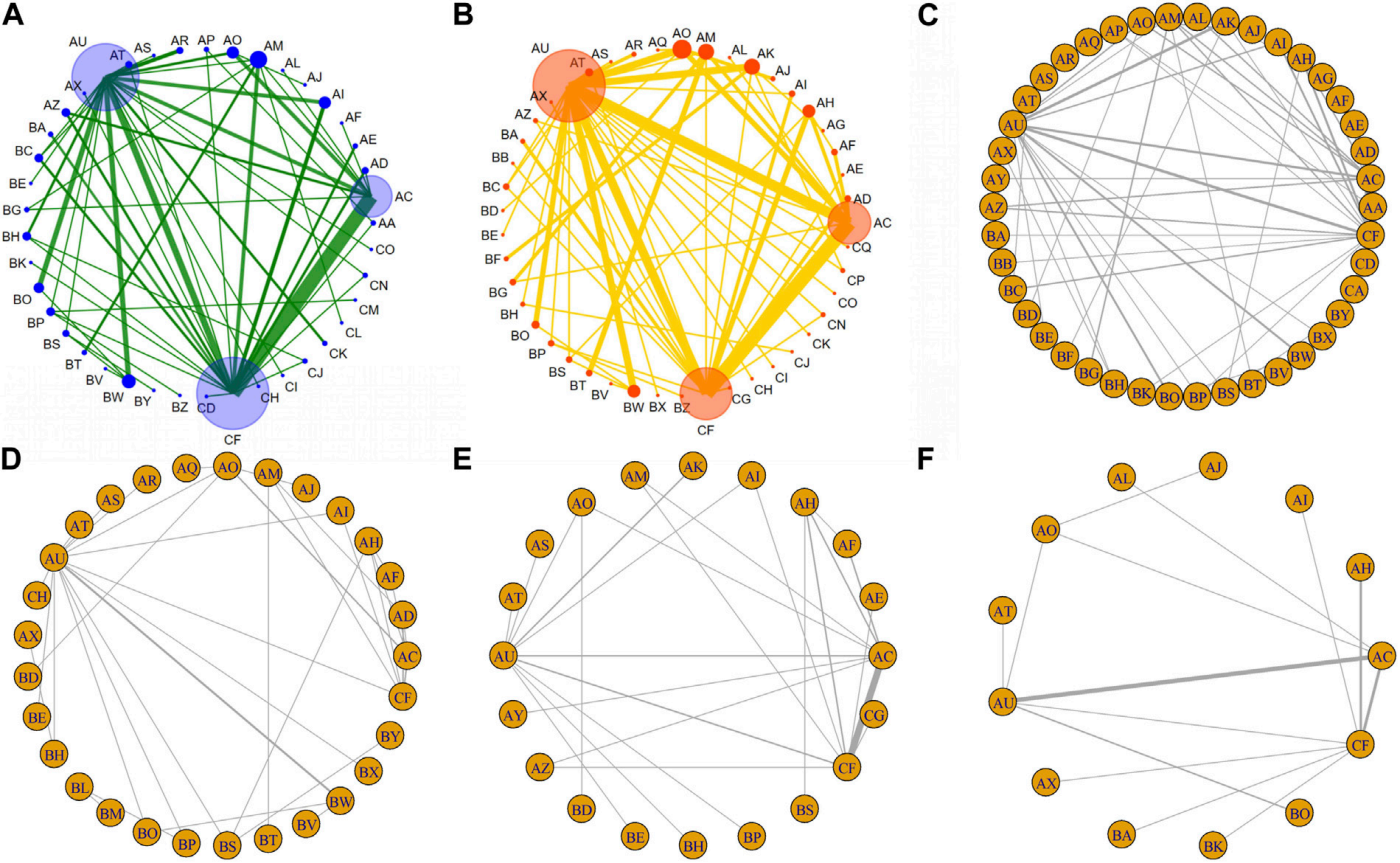
Network diagram comparing treatment outcomes of LA-HNSCC patients with different treatment options. **(A)** ORR. **(B)** ≥3 AEs. **(C)** OS. **(D)** PFS. **(E)** LRC. **(F)** DFS. AA: (Cisplatin + Bleomycin), AB: (Cisplatin + Bleomycin + Methotrexate), AC: AFRT, AD: CCRT(5-Fluorouracil + RT), AE: CCRT(Bleomycin + RT), AF: CCRT(Carboplatin+5-Fluorouracil + AFRT), AG: CCRT(Carboplatin+5-Fluorouracil + Cetuximab + RT), AH: CCRT(Carboplatin+5-Fluorouracil + RT), AI: CCRT(Carboplatin + RT), AJ: (Cetuximab + AFRT), AK: (Cetuximab + RT), AL: CCRT(Cisplatin + 5-Fluorouracil + AFRT), AM: CCRT(Cisplatin + 5-Fluorouracil + RT), AN: CCRT(Cisplatin+5-Fluorouracil + RT)/(Cetuximab + RT), AO: CCRT(Cisplatin + AFRT), AP: CCRT(Cisplatin + Bleomycin + RT), AQ: CCRT(Cisplatin + Cetuximab + AFRT), AR: CCRT(Cisplatin + Gemcitabine + RT), AS: CCRT(Cisplatin + Lapatinb + RT), AT: CCRT(Cisplatin + Nimotuzumab + RT), AU: CCRT(Cisplatin + RT), AV: CCRT(Docetaxel + Cisplatin + 5-Fluorouracil + AFRT), AW: CCRT(Docetaxel + Cisplatin + 5-Fluorouracil + RT), AX: CCRT(Gemcitabine + RT), AY: CCRT(Mitomycin + 5-Fluorouracil + AFRT), AZ: CCRT(Mitomycin + AFRT), BA: CCRT(Mitomycin + Bleomycin + RT), BB: CCRT(Methotrexate + RT), BC: CCRT(Nimotuzumab + RT), BD: CCRT(Panitumumab + AFRT), BE: CCRT(Panitumumab + Cisplatin + RT), BF: CCRT(Pemetrexed + RT), BG: CCRT(Paclitaxel + Cisplatin + RT), BH: CCRT(Paclitaxel + RT), BI: CCRT(Vinblastine + Bleomycin + Methotrexate + RT), BJ: IC(5-Fluorouracil + Cisplatin) + RT, BK: IC(Carboplatin + Teganox)+RT, BL: IC(Carboplatin + Paclitaxel + Cetuximab)+CCRT(Cisplatin + Cetuximab + AFRT), BM: IC(Carboplatin + Paclitaxel + Cetuximab)+CCRT(HU+5-Fluorouracil + Cetuximab + AFRT), BN: IC(Cisplatin + 5-Fluorouracil) + CCRT(Carboplatin + RT), BO: IC(Cisplatin + 5-Fluorouracil) + CCRT(Cisplatin + RT), BP: IC(Cisplatin + 5-Fluorouracil) + RT, BQ: IC(Cisplatin + Bleomycin + Methotrexate) + RT, BR: IC(Docetaxel + Cisplatin + 5-Fluorouracil) + CCRT(Carboplatin + RT), BS: IC(Docetaxel + Cisplatin + 5-Fluorouracil)+ (cetuximab + RT), BT: IC(Docetaxel + Cisplatin + 5-Fluorouracil) + CCRT(Cisplatin + 5-Fluorouracil + RT), BU: IC(Docetaxel + Cisplatin+5-Fluorouracil) + CCRT(Cisplatin + 5-Fluorouracil + RT)/(Cetuximab + RT), BV: IC(Docetaxel + Cisplatin+5-Fluorouracil) + CCRT(Cisplatin + AFRT), BW: IC(Docetaxel + Cisplatin + 5-Fluorouracil) + CCRT(Cisplatin + RT), BX: IC(Docetaxel + Cisplatin+5-Fluorouracil) + CCRT(Docetaxel/Carboplatin + RT), BY: IC(Docetaxel + Cisplatin + Cetuximab)+ (cetuximab + RT), BZ: IC(Pemetrexed + Cisplatin)+RT, CA: IC(Methotrexate)+RT, CB: IC(Paclitaxel + Cisplatin) + CCRT(Cisplatin + 5-Fluorouracil + RT), CC: IC(Paclitaxel + Cisplatin) + CCRT(Cisplatin + Capecitabine + RT), CD: IC(Vincristine + Methotrexate)+RT, CE: IC(Vinblastine + Bleomycin + Methotrexate)+RT, CF:RT, CG: CCRT(Mitomycin + RT), CH: CCRT(Erlotinib + Cisplatin + RT), CI: CCRT(Cisplatin + Capecitabine + RT), CJ: CCRT(Gemcitabine + RT), CK: CCRT(Vinorelbine + RT), CL: CCRT(Raloxifen + Cisplatin + RT), CM: IC(Cisplatin + 5-Fluorouracil)+CCRT(Cisplatin + 5-Fluorouracil + RT), CN: IC(Cisplatin)+RT, CO: IC(Lapatinb) + CCRT(Cisplatin + RT), CP: CCRT(Avelumab + Cetuximab + RT), CQ: CCRT(Avelumab + Cisplatin + RT).

**FIGURE 3:**
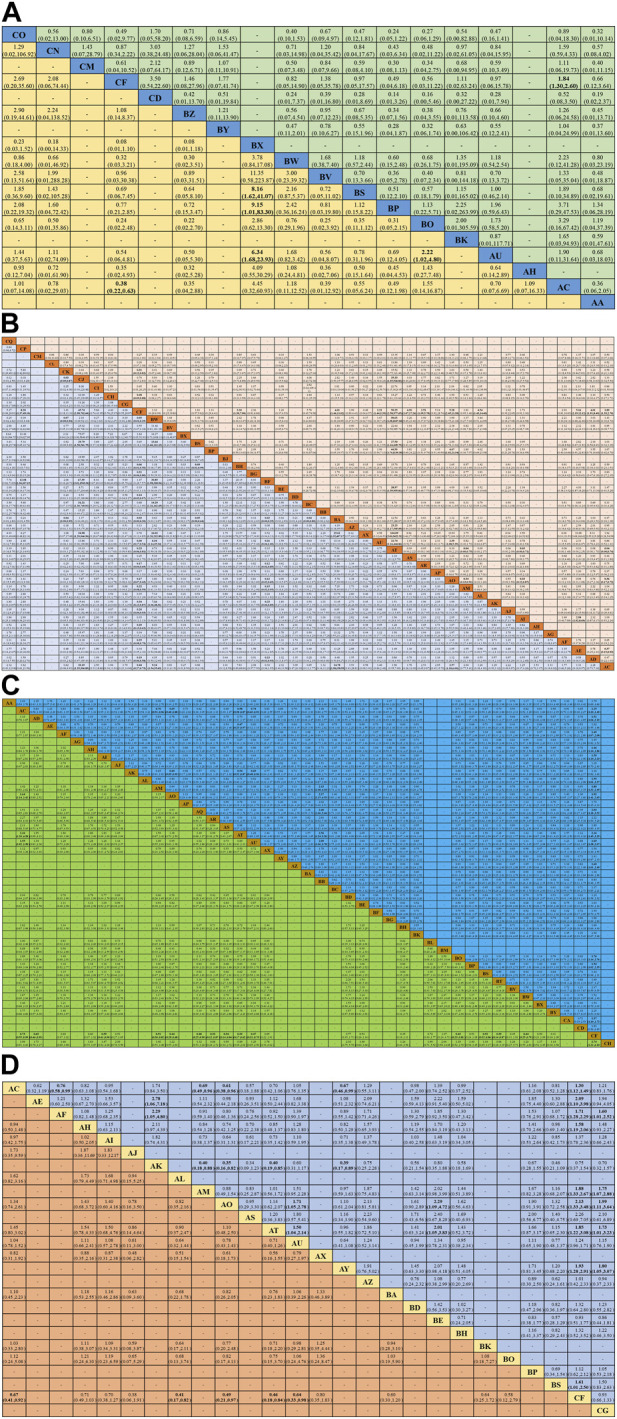
Summary of network meta-analysis. **(A)** Combined odds ratio (95% onfidence interval) of ORR (upper triangle) and ≥3 AEs (lower triangle) for IC combined with CCRT regimens; **(B)** Combined hazard ratio (95% onfidence interval) of ORR (upper triangle) and ≥3 AEs (lower triangle) for systemic treatment with concurrent RT regimens; **(C)** Combined hazard ratio (95% confidence interval) for OS (upper triangle) and PFS (lower triangle); **(D)** Combined hazard ratio (95% confidence interval) for LRC (upper triangle) and DFS (lower triangle). The data in each cell is a risk ratio or hazard ratio (95% confidence interval) that is used to compare the column *versus* the row. Statistically significant results are shown in bold.

There were 86 RCTs trials reporting ORR, including 12 IC combined with CCRT regimens and 27 systemic treatments with concurrent RT regimens (Figure 2A). The results showed that IC combined with CCRT regimens failed to achieve a significant ORR benefit compared with RT alone (Figure 3A). In contrast, 11 clinical trials compared conventional fractionated RT with accelerated fractionated RT (AFRT). The results showed that AFRT had a better ORR than RT (Figure 3B; Supplementary Figure S4). Similarly, CCRT (cisplatin + AFRT) had a significantly better ORR than CCRT (cisplatin + RT) (Figure 3B). All 12 systemic treatments with concurrent RT regimens were significantly more effective than RT alone (Figure 3B), including CCRT (5-Fluorouracil + RT), CCRT (bleomycin + RT), CCRT (cisplatin+5-Fluorouracil + AFRT), CCRT (cisplatin + AFRT), CCRT (cisplatin + gemcitabine + RT), CCRT (cisplatin + lapatinib + RT), CCRT(cisplatin + nimotuzumab + RT), CCRT (cisplatin + RT), CCRT(erlotinib + cisplatin + RT), CCRT(mitomycin C + bleomycin + RT), CCRT(vinorelbine + RT), CCRT(paclitaxel + RT). Meanwhile, CCRT (cisplatin + nimotuzumab + RT) showed a better ORR than conventional CCRT regimens, such as CCRT (cisplatin + RT), CCRT (gemcitabine + RT), CCRT (mitomycin C + AFRT), CCRT (panitumumab + cisplatin + RT), and so on.

**FIGURE 4:**
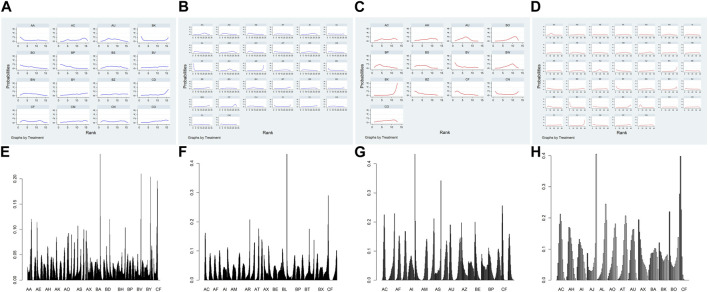
Bayesian sequencing of the efficacy of different treatment regimens in LA-HNSCC patients. **(A)** ORR of IC combined with CCRT regimens; **(B)** ORR of systemic treatment with concurrent RT regimens; **(C)** ≥ 3AEs of IC combined with CCRT regimens; **(D)** ≥ 3AEs of systemic treatment with concurrent RT regimens; **(E)** OS; **(F)** PFS; **(G)** LRC; **(H)** DFS. The sorting graph is described based on bayesian sorting results in Supplementary Table S3.

Figures 4A,B; Supplementary Table S3 shows the Bayesian ranking results for the different treatment regimens. SUCRA values were used to rank the different treatment regimens, with higher values indicating higher efficiency (Supplementary Table S4). Similar to the consistency model results, almost all systemic treatments with concurrent RT regimens had better ORR than RT alone. In addition, within the IC combined with CCRT regimens, only IC (docetaxel + cisplatin+5-Fluorouracil) combined with cetuximab and concurrent with RT regimen showed a better ORR compared to RT alone. Meanwhile, CCRT (cisplatin + nimotuzumab + RT) had the highest SUCRA values, while nimotuzumab concurrent with RT regimen was superior to most of CCRT regimens.

### AEs

There were 86 RCT trials reporting ≥3 AEs, including 9 IC combined with CCRT treatment regimens and 35 systemic treatments with concurrent RT regimens (Figure 2B). The results demonstrated that AFRT may occur with higher proportions of ≥3 AEs compared to RT with statistically significant differences (Figure 3B; Supplementary Figure S5). IC (docetaxel + cisplatin+5-Fluorouracil) combined with CCRT (docetaxel/carboplatin + RT) regimen was associated with a higher proportion of ≥3 AEs than IC (docetaxel + cisplatin+5-Fluorouracil) combined with cetuximab concurrent with RT and IC (cisplatin+5-Fluorouracil) combined with RT (Figure 3A). Compared to RT alone, 15 systemic treatments with concurrent RT regimens showed significantly more ≥3 AEs, including CCRT (5-Fluorouracil + RT), CCRT (avelumabe + cetuximab + RT), CCRT (carboplatin+5-Fluorouracil + AFRT), CCRT (carboplatin+5-Fluorouracil + RT), CCRT (cisplatin+5-Fluorouracil + RT), CCRT (cisplatin + AFRT), CCRT (cisplatin + gemcitabine + RT), CCRT (cisplatin + nimotuzumab + RT), CCRT (cisplatin + RT), CCRT (mitomycin C + bleomycin + RT), CCRT (panitumumab + AFRT), CCRT (paclitaxel + cisplatin + RT), CCRT(paclitaxel + RT), and cetuximab concurrent with RT or AFRT regimens. Notably, CCRT (nimotuzumab + RT) showed a statistically significant lower ≥3 AE compared to various common treatment regimens, with statistically significant differences, including CCRT (cisplain + capecitabine + RT), CCRT (mitomycin C + bleomycin + RT) and IC(docetaxel + cisplain+5-Fluorouracil) combined with CCRT(cisplain + RT).

The results of the Bayesian ranking of ≥3 AEs for the different treatment regimens are shown in Figures 4C,D; Supplementary Table S3. SUCRA values were used to rank the different treatment regimens, with higher values indicating lower ≥3 AEs (Supplementary Table S4). Like the consistency model results, almost all systemic treatments with concurrent RT regimens and IC combined with CCRT treatment regimens exhibited higher ≥3 AEs compared with RT alone. The ≥3 AEs of IC (docetaxel + cisplatin+5-Fluorouracil) combined with CCRT (docetaxel/carboplatin + RT) was significantly higher than that of almost all the other treatment regimens. Nimotuzumab concurrent with RT regimen had the highest SUCRA values, followed by RT alone.

### OS

There were 93 RCTs reporting OS, including 36 systemic treatments with concurrent RT regimens and 17 IC combined with CCRT regimens (Figure 2C). Of these, 15 treatment regimens showed more favorable OS than RT alone: AFRT, CCRT (5-Fluorouracil + RT), CCRT (carboplatin+5-Fluorouracil + AFRT), CCRT (carboplatin+5-Fluorouracil + RT), CCRT (carboplatin + RT), CCRT (cisplatin+5-Fluorouracil + AFRT), CCRT (cisplatin+5-Fluorouracil + RT), CCRT (cisplatin + AFRT), CCRT (cisplatin + cetuximab + AFRT), CCRT (cisplatin + nimotuzumab + RT), CCRT (cisplatin + RT), CCRT (mitomycin C + AFRT), IC (cisplatin+5-Fluorouracil) combined with CCRT (cisplatin + RT), IC (cisplatin+5-Fluorouracil) combined with RT, and IC (methotrexate) combined with RT. Notably, CCRT (cisplatin + nimotuzumab + RT) showed a statistically significant survival advantage compared to AFRT, CCRT (bleomycin + RT), and cetuximab concurrent with RT regimens. CCRT (cisplatin + RT), a commonly used CCRT regimen recommended by the NCCN guidelines, showed better OS than AFRT and cetuximab concurrent with RT regimens (Figure 3C).

Figure 4E; Supplementary Table S3 shows the Bayesian ranking results of the OS for different treatment regimens. SUCRA values were used to rank the different treatment regimens, with lower scores indicating higher OS (Supplementary Table S4). The results showed that CCRT (cisplatin + nimotuzumab + RT) had the lowest SUCRA scores and the best OS. Platinum-based CCRT regimens such as CCRT (cisplatin + AFRT), CCRT (cisplatin+5-Fluorouracil + RT), CCRT (cisplatin + gemcitabine + RT), and CCRT (cisplatin + RT) may provide better OS. Furthermore, conventional fractionated RT had higher SUCRA values and lower OS than almost all treatment regimens.

### PFS

PFS was reported in 38 RCTs, including 22 systemic treatments with concurrent RT regimens and 15 IC combined with CCRT regimens (Figure 2D). Consistent with the OS results, 15 combination regimens showed better PFS than RT, including AFRT, CCRT (5-Fluorouracil + RT), CCRT (carboplatin + RT), CCRT (cisplatin+5-Fluorouracil + RT), CCRT (cisplatin + AFRT), CCRT (cisplatin + cetuximab + AFRT), CCRT (cisplatin + gemcitabine + RT), CCRT (cisplatin + lapatinib + RT), CCRT (cisplatin + nimotuzumab + RT), CCRT (cisplatin + RT), CCRT (erlotinib + cisplatin + RT), IC (cisplatin+5-Fluorouracil) combined with CCRT (cisplatin + RT), IC (docetaxel + cisplatin+5-Fluorouracil) combined with CCRT (cisplatin+5-Fluorouracil + RT), IC (docetaxel + cisplatin+5-Fluorouracil) combined with CCRT (cisplatin + RT), IC (docetaxel + cisplatin+5-Fluorouracil) combined with cetuximab concurrent RT,. Furthermore, CCRT (cisplatin + AFRT), CCRT (cisplatin + nimotuzumab + RT), and CCRT (cisplatin + RT) showed significantly better PFS than AFRT (Figure 3C).

Figure 4F; Supplementary Table S3 shows the results of the Bayesian ranking of PFS for different treatment regimens. SUCRA values were used to rank the different treatment regimens, with lower scores indicating higher PFS (Supplementary Table S4). Consistently, CCRT (cisplatin + nimotuzumab + RT) had the lowest SUCRA values and probably the best PFS. Almost all CCRT regimens had better PFS than the RT and AFRT regimens.

### LRC

There were 32 RCTs reporting LRC, including 22 treatment regimens (Figure 2E). The results showed that AFRT, CCRT (bleomycin + RT), CCRT (carboplatin+5-Fluorouracil + AFRT), CCRT (carboplatin+5-Fluorouracil + RT), CCRT (cisplatin+5-Fluorouracil + RT), CCRT (cisplatin + AFRT), CCRT (mitomycin C+5-Fluorouracil + AFRT), and IC (docetaxel + cisplatin+5-Fluorouracil) combined with cetuximab concurrent with RT regimens showed better local control compared than RT alone. CCRT (mitomycin C + RT), CCRT (carboplatin+5-Fluorouracil + AFRT), CCRT (cisplatin+5-Fluorouracil + RT), CCRT (cisplatin + AFRT), CCRT (cisplatin + nimotuzumab + RT), and CCRT (mitomycin C+5-Fluorouracil + AFRT) showed better LRC. CCRT (cisplatin + AFRT), the gold standard recommended by the NCCN for LA-HNSCC patients, showed better local tumor control than RT, AFRT, CCRT (cisplatin + RT), CCRT (panitumumab + cisplatin + RT), CCRT (mitomycin C + RT), and cetuximab concurrent with RT regimens (Figure 3D).

Figure 4G; Supplementary Table S3 shows the Bayesian ranking results of LRC for different treatment regimens. SUCRA values were used to rank the different treatment regimens, with lower scores indicating better LRC (Supplementary Table S4). It is worth noting that CCRT (cisplatin + AFRT) had the lowest score and was most likely to achieve the highest LRC. In addition, RT and cetuximab concurrent with the RT regimen ranked first.

### DFS

DFS was reported in 26 RCTs that included 19 treatment regimens (Figure 2F). The results showed that AFRT, CCRT (cisplatin+5-Fluorouracil + AFRT), CCRT (cisplatin + AFRT), CCRT (cisplatin + nimotuzumab + RT), and CCRT (cisplatin + RT) showed statistically significant differences in DFS compared to RT (Figure 3D). Bayesian ranking results showed that CCRT (cisplatin+5-Fluorouracil + AFRT) and CCRT (cisplatin + nimotuzumab + RT) have the lowest DFS and the RT rank first, respectively (Figure 4H; Supplementary Tables S3–S5).

### DSS

DSS was reported in only 10 RCTs comprised of 9 treatment regimens (Supplementary Figure S6). There were no statistical differences among the treatment regimens after fitting the consistency model (Supplementary Figure S7). Bayesian ranking results showed that concurrent RT and cetuximab with an RT regimen had the lowest possible DSS (Supplementary Figure S8; Supplementary Tables S3–S4). CCRT (cisplatin+5-Fluorouracil + AFRT) and CCRT (cisplatin+5-Fluorouracil + RT) showed the lowest SUCRA values and a better DSS.

In addition, the consistency of results showed that the differences between all the direct and indirect comparisons were not statistically significant, shown in Supplementary Figure S9; Supplementary Figure S10. Further, the consistency model fit satisfactorily. Funnel plots were drawn to assess publication bias (Supplementary Figure S11; Supplementary Figure S12).

## Discussion

In recent years, remarkable progress has been made in LA-HNSCC treatment. Nevertheless, the treatment options for LA-HNSCC patients remain a complex topic. Therefore, we conducted a systematic review and network meta-analysis of multiple treatment regimens for LA-HNSCC. A total of 126 RCTs were included in this network meta-analysis to summarize the evidence on the efficacy and safety of different treatment options.

To date, this is the most comprehensive review of the safety and efficacy of full treatment options for LA-HNSCC patients. The main study findings are summarized as follows: CCRT (cisplatin + nimotuzumab + RT) and nimotuzumab concurrent with RT have significant advantages in both efficacy and long-term survival compared with various conventional LA-HNSCC treatment regimens, including platinum-based CCRT regimens (including cisplatin and carboplatin). At the same time, it was surprising that CCRT (cisplatin + nimotuzumab + RT) and nimotuzumab concurrent with RT did not cause a higher proportion of ≥3 AEs than most combination regimens. This suggests that CCRT (cisplatin + nimotuzumab + RT) and nimotuzumab combined with RT may be the most promising treatment option for LA-HNSCC. The efficacy and safety advantages of CCRT (cisplatin + nimotuzumab + RT) and nimotuzumab concurrent with RT regimens warrant a higher-level recommendation by the NCCN guidelines. Our results provide further directions for using nimotuzumab in LA-HNSCC patients. IC combined with CCRT failed to show a statistically significant difference in efficacy compared to RT alone while incurring a significantly higher risk of AEs, suggesting that IC combined with CCRT regimens should be carefully chosen for LA-HNSCC patients. In addition, AFRT showed higher efficacy than RT, despite being associated with significantly increased AEs. Cetuximab concurrent with RT regimen as an alternative to platinum-based chemotherapy did not show a better advantage in OS, PFS, and DSS. In addition, cetuximab concurrent with RT regimen was associated with a higher rate of ≥3 AEs than RT, suggesting that the recommendation grade in future NCCN guidelines needs to be adjusted or removed.

### Study strengths and limitations

This study provides a comprehensive analysis of the effectiveness and long-term survival of 78 combination treatment regimens by investigating six outcome indicators, including ORR, OS, PFS, LRC, DFS, and DSS. Before starting this study, an extensive and detailed search strategy was developed. The included data were comprehensive and included results from unofficially published data. The patient population and definition of outcome indicators included in the original studies were assessed to ensure the transferability of the study. Furthermore, an inconsistency examination was conducted to assess whether the direct and indirect comparisons were consistent. More importantly, the generally low I^2^ values indicated acceptable agreement, suggesting satisfactory confidence in our study.

Nevertheless, systematic reviews and network meta-analyses have certain limitations. First, not all outcome indicators from the original studies were analyzed, leading to the possibility that the strengths of some treatment regimens for a particular indicator may have been overlooked. Second, network meta-analyses simultaneously perform direct and indirect comparisons of multiple treatment regimens, but some studies contained only indirect comparisons between regimens. Indirect comparisons increase uncertainty for results assessment. On the other hand, the validity of indirect comparisons depends on the intrinsic validity and their similarity of the trials involved, so more indirect comparisons may reduce the reliability of the results. Therefore, the indirect comparison results should be cautiously applied in clinical practice. In the future, clinical trials which generated grade 1 evidence should be given increased attention. The SUCRA values obtained from Bayesian analysis reflected the ranking among multiple treatment regimens, but it was impossible to specifically identify the statistically significant differences, which resulted in some uncertainty in the results. Furthermore, most of the included original studies did not clearly articulate the use of blinding and allocation concealment, leading to a decline in methodological quality. Additionally, a total of 126 RCTs from 1975 to 2021 were included in this study, and the updates of tumor staging criteria and treatment regimens during this period increased the heterogeneity of the included studies. In addition, this study did not limit the patient race and tumor subregion from the original studies, which limits the generalizability of the results. Undoubtedly, standardizing the included patients will be vital for comparing and evaluating treatment options in future clinical trials.

### Study implications

This study aimed to determine the best choice of clinical treatment options by providing a comprehensive review of multiple treatment regimens that are currently applied to LA-HNSCC. We also evaluated treatment efficacy and safety by applying seven outcome indicators.

Previous studies confirmed that CCRT increases survival in LA-HNSCC patients, with a 6.5% increase in 5-year survival and significantly reduced local failure rates (Pignon et al., 2009; Lacas et al., 2021). The RTOG91-11 trial also confirmed that CCRT is the most effective method for local control and organ preservation in LA-HNSCC patients (Forastiere et al., 2013). High-dose platinum combined with RT is the accepted standard treatment regimen for improved OS of LA-HNSCC patients (Adelstein et al., 2003). In this study, we demonstrate that nimotuzumab-based CCRT regimens have higher efficacy and are not accompanied by a higher incidence of AEs than conventional CCRT regimens while potentially achieving better long-term survival. Nimotuzumab is safer than cetuximab, which has a higher rate of rash, probably because nimotuzumab is a fully-humanized monoclonal antibody, whereas cetuximab is a human-mouse chimeric antibody (Bonner et al., 2010; Xu et al., 2017). Although this finding needs to be confirmed by higher-quality RCT studies, it may provide a direction for future clinical trials. HNSCC is transforming from uniform intensive treatment focused simply on improving survival to individualized treatment based on a combination of biology, biomarkers, and immunotherapy. In addition, many ongoing RCTs are exploring the efficacy and safety of immunotherapy in LA-HNSCC (Weiss et al., 2020; Zech et al., 2020). Thus, we may obtain more evidence to support the efficacy of immunotherapy in the future.

Previously, IC was reported to reduce the risk of distant metastases (Marta et al., 2015; Li et al., 2021). IC has also been proposed to improve OS in LA-HNSCC patients (Bossi et al., 2014). In patients with unresectable LA-HNSCC, sequential treatment of IC and CCRT has been explored as an intensive treatment approach (Haddad et al., 2013; Cohen et al., 2014). However, the efficacy of IC remains controversial. IC (docetaxel + cisplatin+5-Fluorouracil) combined with CCRT showed no significant improvement in OS compared to CCRT alone (Hitt et al., 2014). This study shows that IC combined with CCRT regimens does not provide a better efficiency and survival benefit than RT alone but is associated with significantly higher AEs. Therefore, the feasibility of IC in LA-HNSCC patients requires further investigation. IC may be reasonably assessed as a screening method, with further options for intensive RT or CCRT depending on the patient’s response to IC (Chen et al., 2017; Marur et al., 2017). As expected, CCRT regimens had higher efficiency and better survival trends compared to RT alone, particularly nimotuzumab-based and conventional platinum-based CCRT regimens. In patients who cannot tolerate platinum-based chemotherapy, cetuximab combined with RT improves survival in LA-HNSCC patients compared with RT alone (Bonner et al., 2006). However, cetuximab concurrent with RT regimen did not show the desired benefit in this study, suggesting that this regimen should be carefully applied in future.

## Conclusion

In this systematic review and network meta-analysis, we simultaneously evaluated the effectiveness and safety of various treatment regimens currently used for LA-HNSCC. Thus far, this study is the most comprehensive summary of treatment options available for LA-HNSCC patients. The results present the benefits of nimotuzumab-based systemic therapy regimens combined with RT in both effectiveness and long-term survival, suggesting their potential for LA-HNSCC treatment. This study reinforces the absolute benefits of AFRT in terms of both efficacy and prognosis compared to conventional fractionated RT and provides a reference for further selecting clinical RT regimens. In addition, we observed no significant improvement of cetuximab in combination with RT compared to RT alone. However, cetuximab combined with RT is accompanied by more significant AEs, suggesting that cetuximab concurrent with RT regimen needs to be evaluated in further large-scale trials.

## Abbreviations

HNCs: Head and neck cancers; LA-HNSCC: Locally advanced head and neck squamous cell carcinoma; RT: radiotherapy; EGFR: epidermal growth factor receptor; FDA: Food and Drug Administration; LRC: local control; OS: overall survival; IC: induction chemotherapy; ORR: objective response rate; PFS: progression-free survival; PRISMA: preferred reporting items for systematic review and meta-analyses, RCTs: randomized controlled trials, DFS: disease-free survival, DSS: disease-specific survival, HR: hazard ratio, CI: confidence interval, OR: odds ratio, CCRT: concurrent radio-chemotherapy, SUCRA: surface under the cumulative ranking, NCCN: National Comprehensive Cancer Network, DIC: deviance information criteria, MCMC: Markov chain Monte Carlo, AE: adverse events.
