# Supplementary figures and images for: Locally advanced head and neck squamous cell carcinoma treatment efficacy and safety: a systematic review and network meta-analysis
Source: Front Pharmacol. 2023 Sep 19;14:1269863. doi: 10.3389/fphar.2023.1269863 (PMC10546034; doi:10.3389/fphar.2023.1269863)

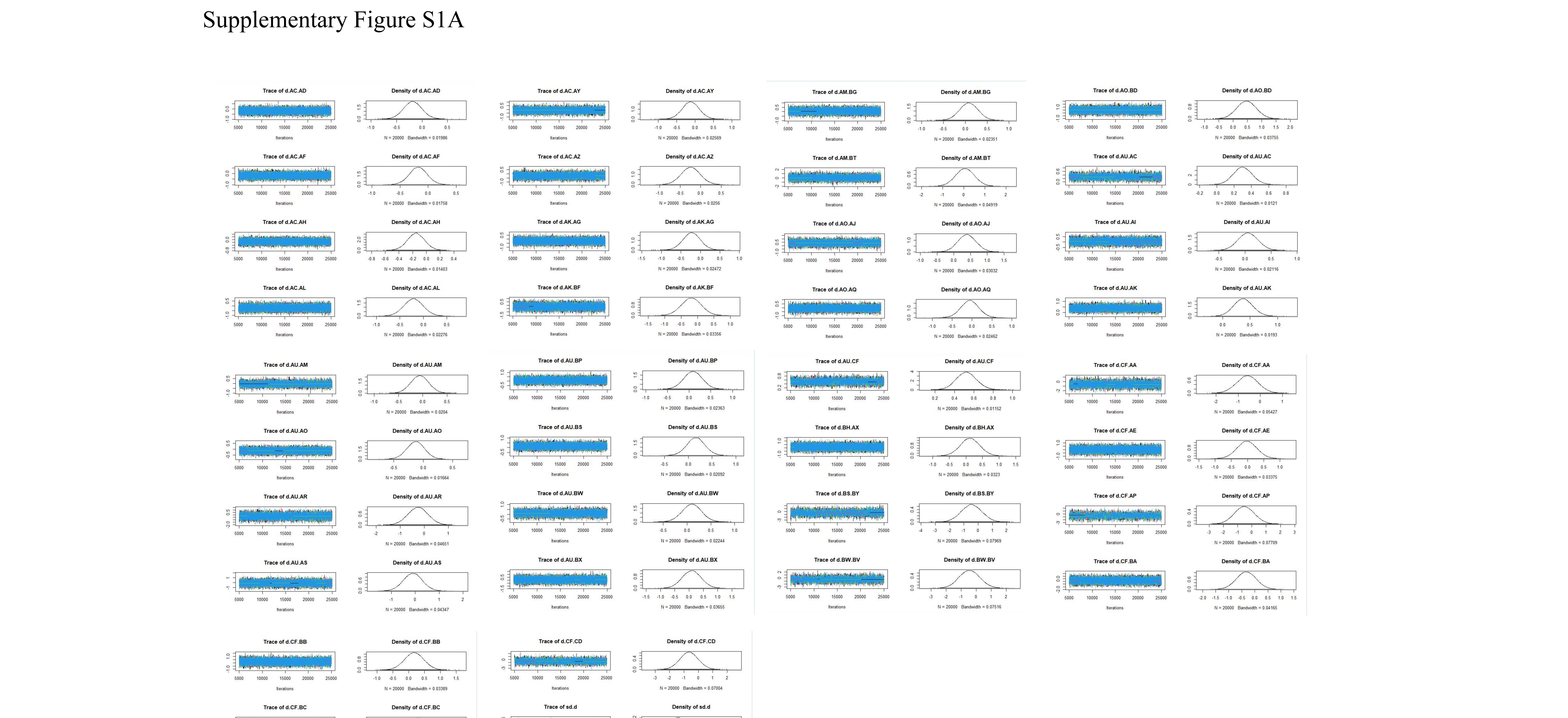

Supplement: Supplementary file 1 [file DataSheet1.zip › Supplementary figures/Supplementary figures_01A.jpg]

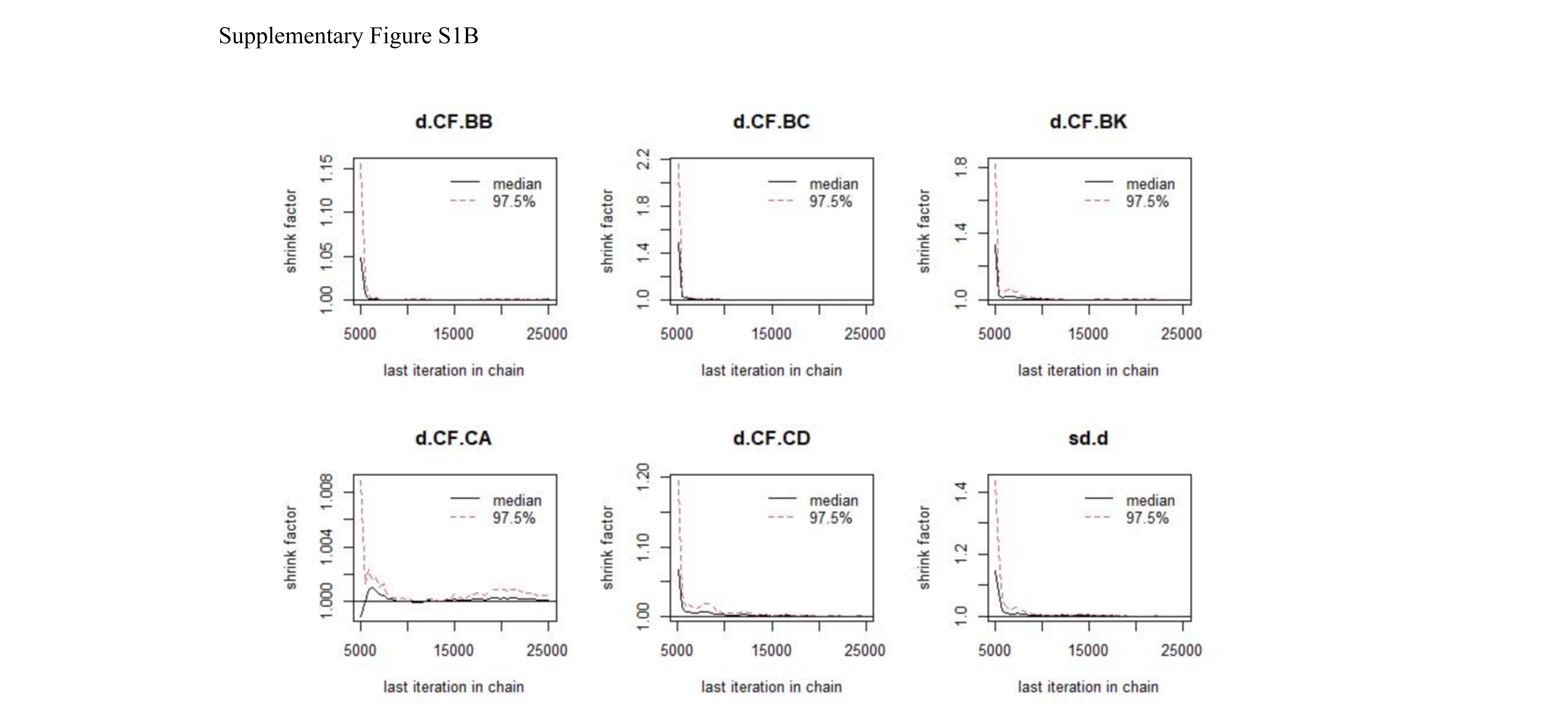

Supplement: Supplementary file 1 [file DataSheet1.zip › Supplementary figures/Supplementary figures_01B.jpg]

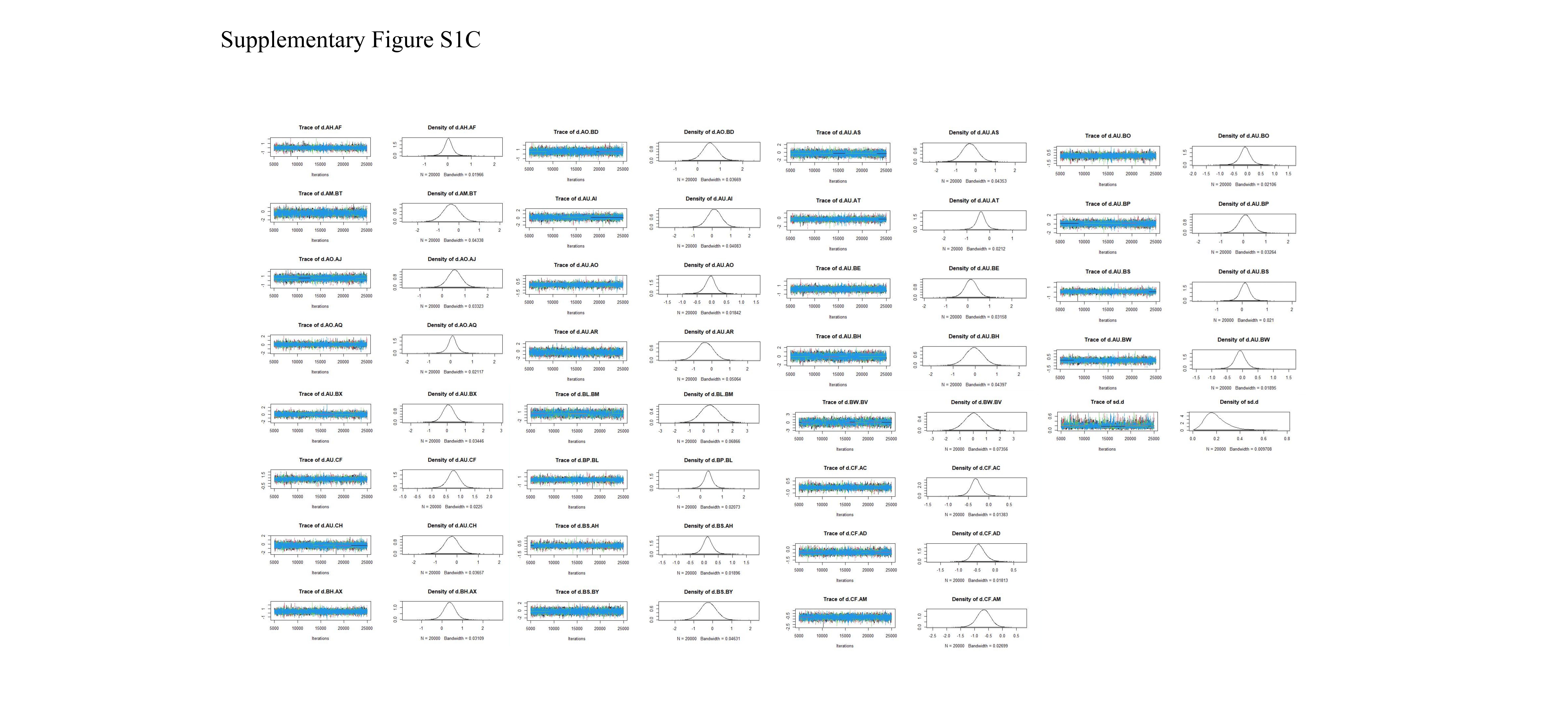

Supplement: Supplementary file 1 [file DataSheet1.zip › Supplementary figures/Supplementary figures_01C.jpg]

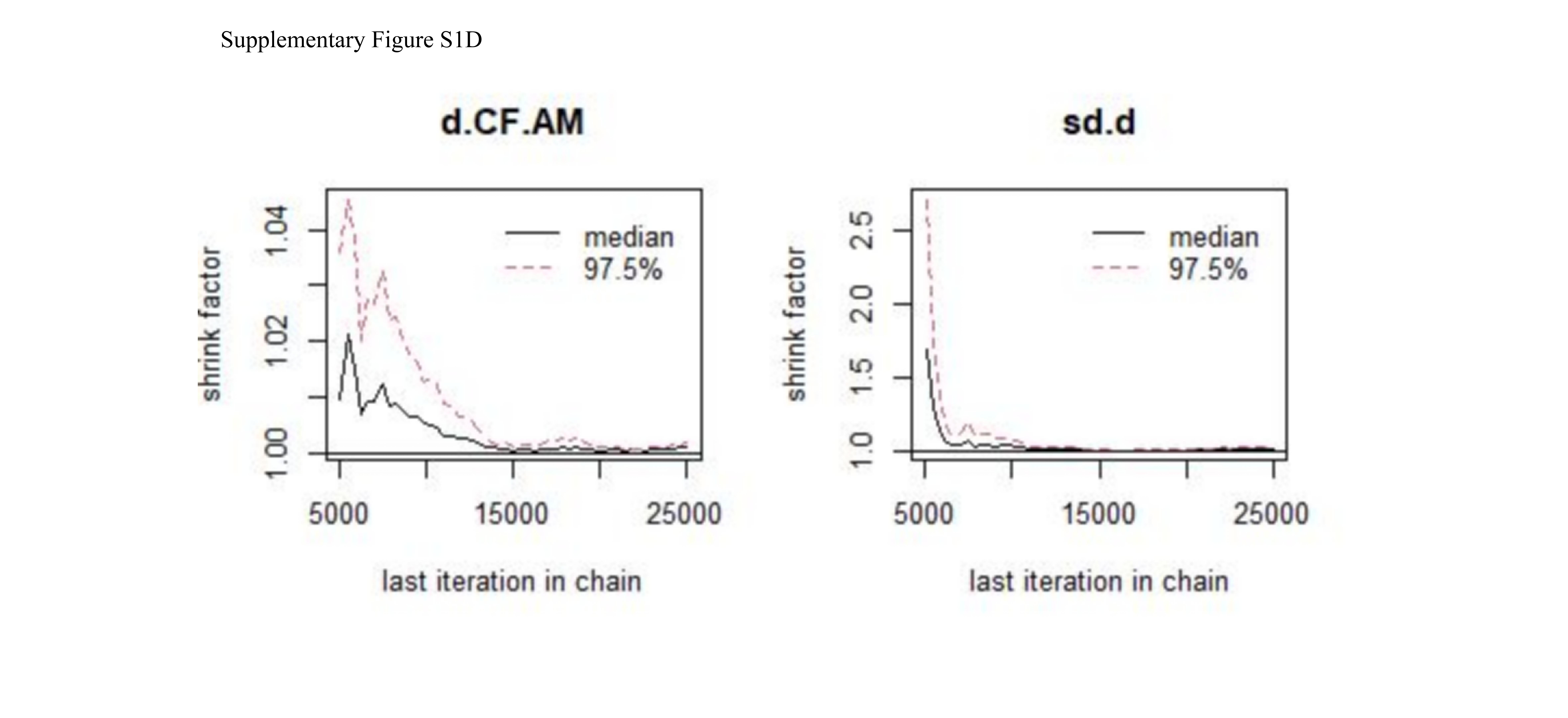

Supplement: Supplementary file 1 [file DataSheet1.zip › Supplementary figures/Supplementary figures_01D.jpg]

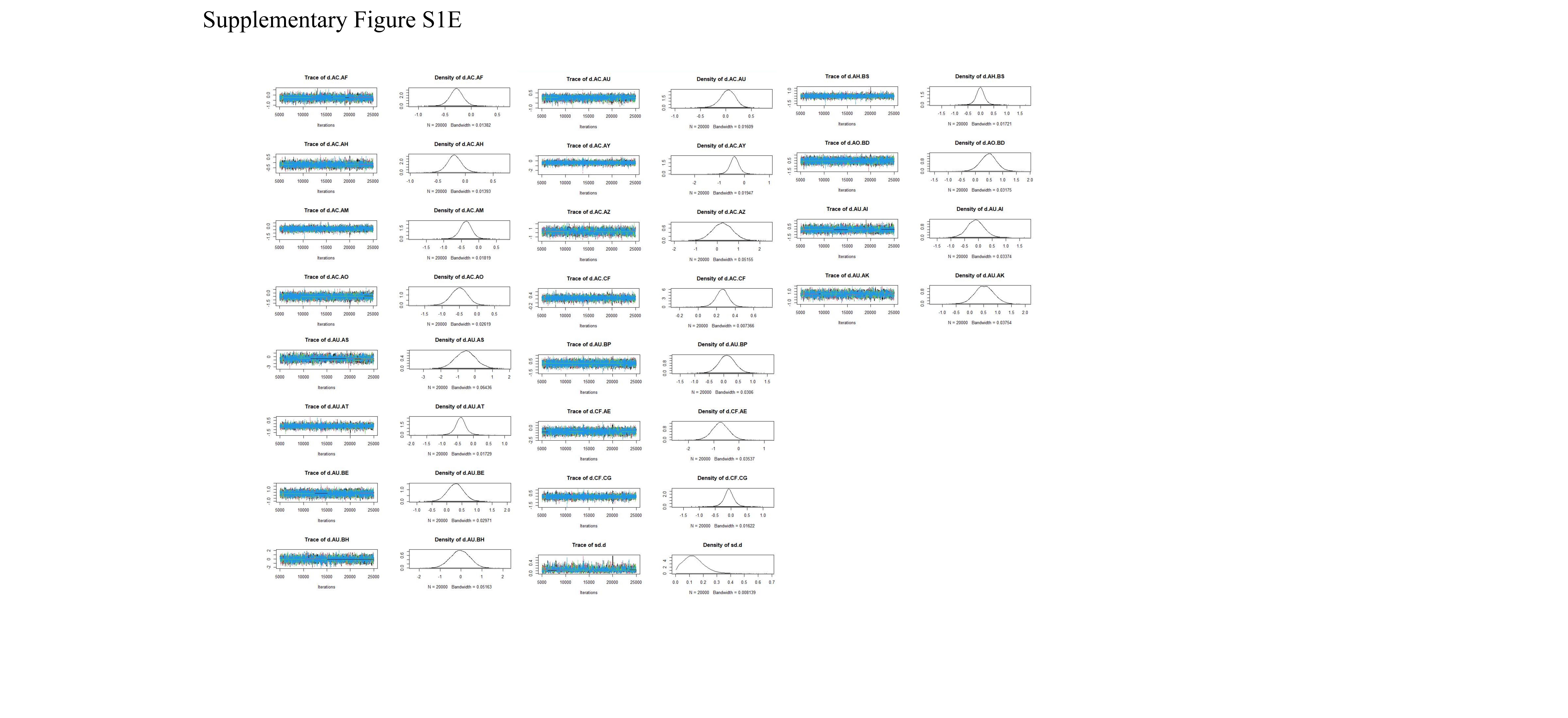

Supplement: Supplementary file 1 [file DataSheet1.zip › Supplementary figures/Supplementary figures_01E.jpg]

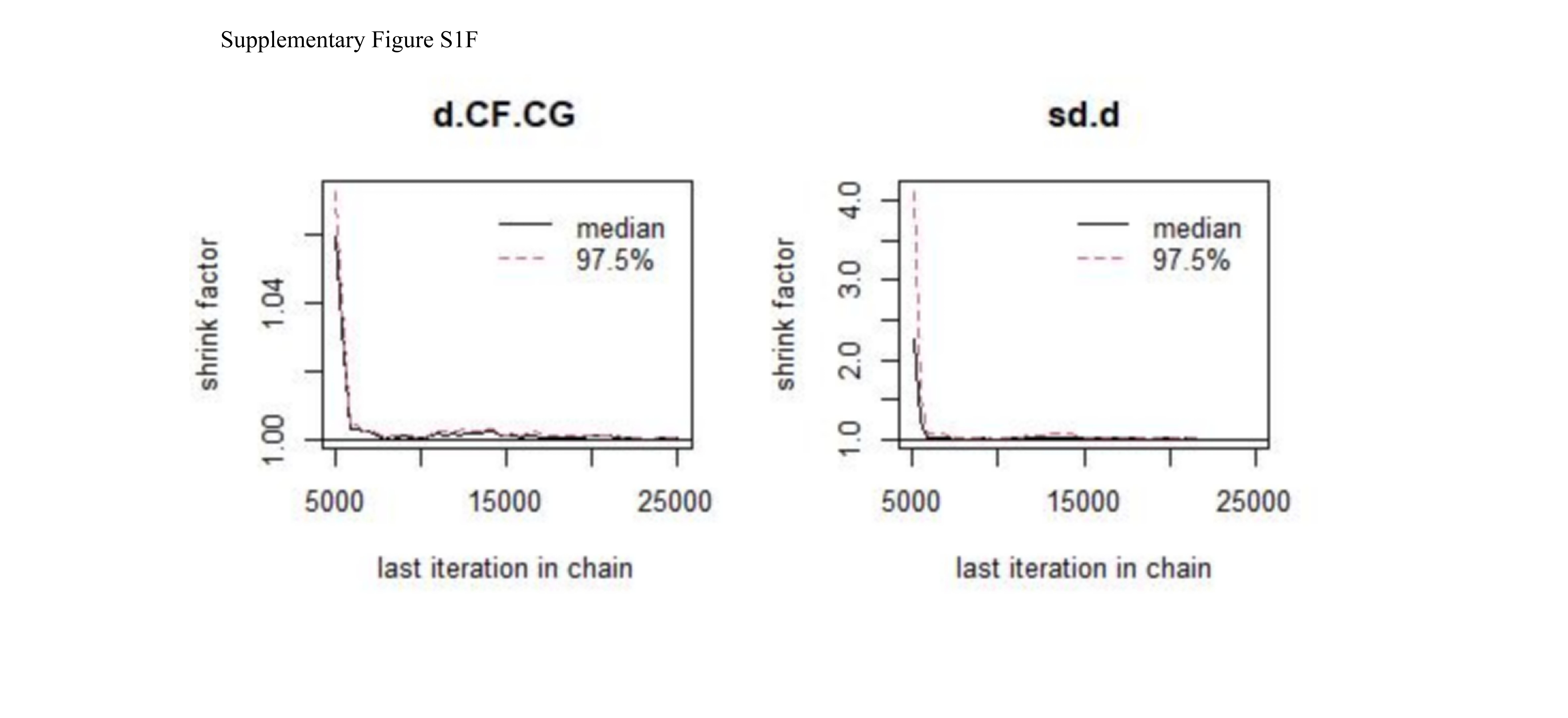

Supplement: Supplementary file 1 [file DataSheet1.zip › Supplementary figures/Supplementary figures_01F.jpg]

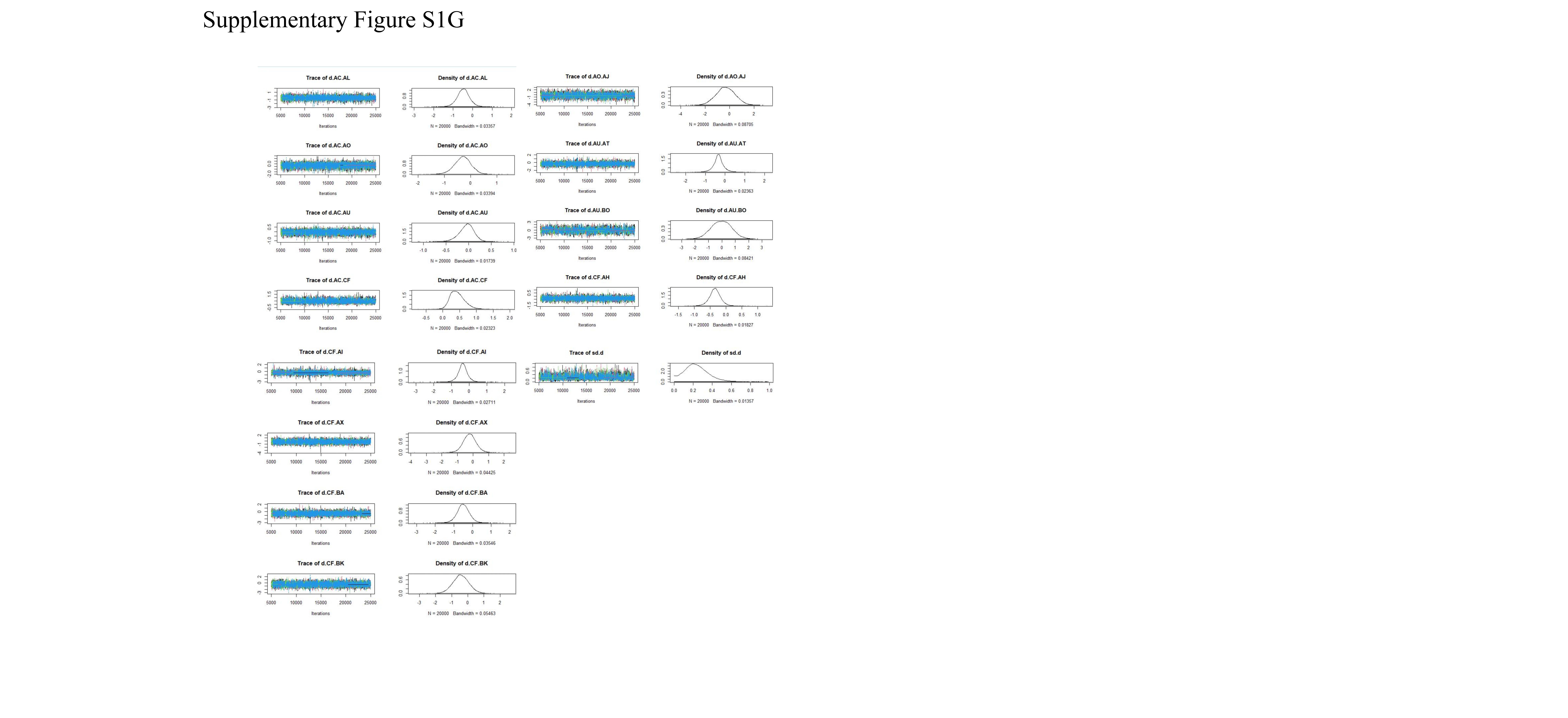

Supplement: Supplementary file 1 [file DataSheet1.zip › Supplementary figures/Supplementary figures_01G.jpg]

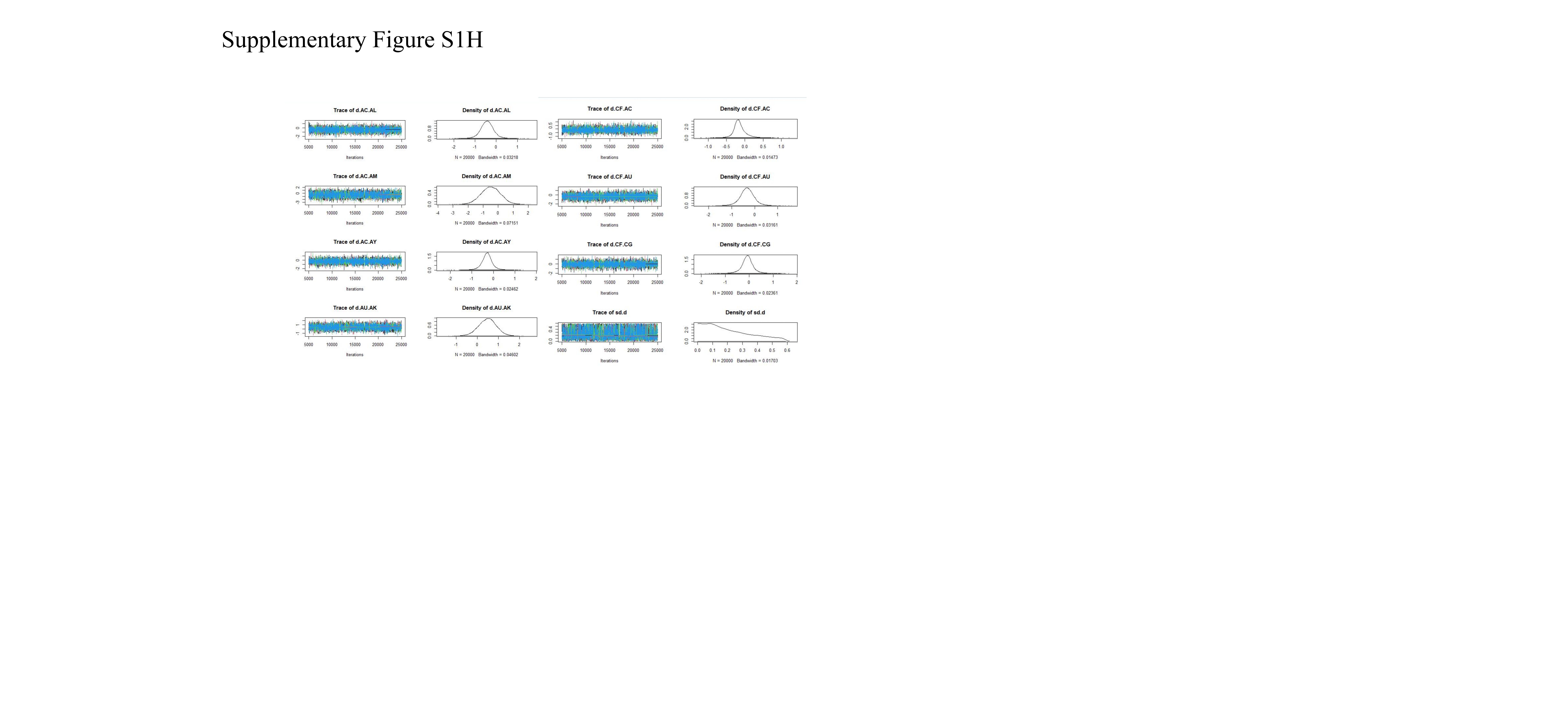

Supplement: Supplementary file 1 [file DataSheet1.zip › Supplementary figures/Supplementary figures_01H.jpg]

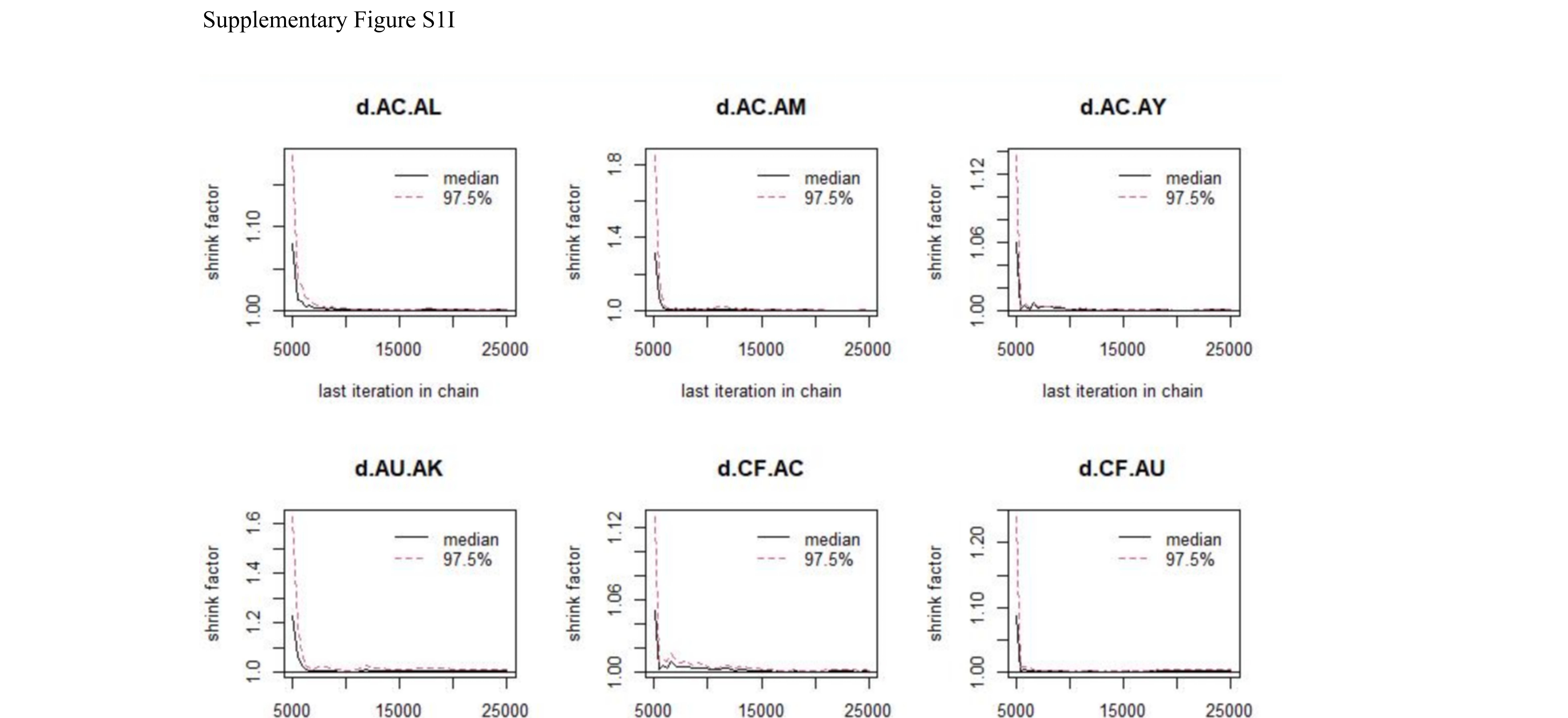

Supplement: Supplementary file 1 [file DataSheet1.zip › Supplementary figures/Supplementary figures_01I.jpg]

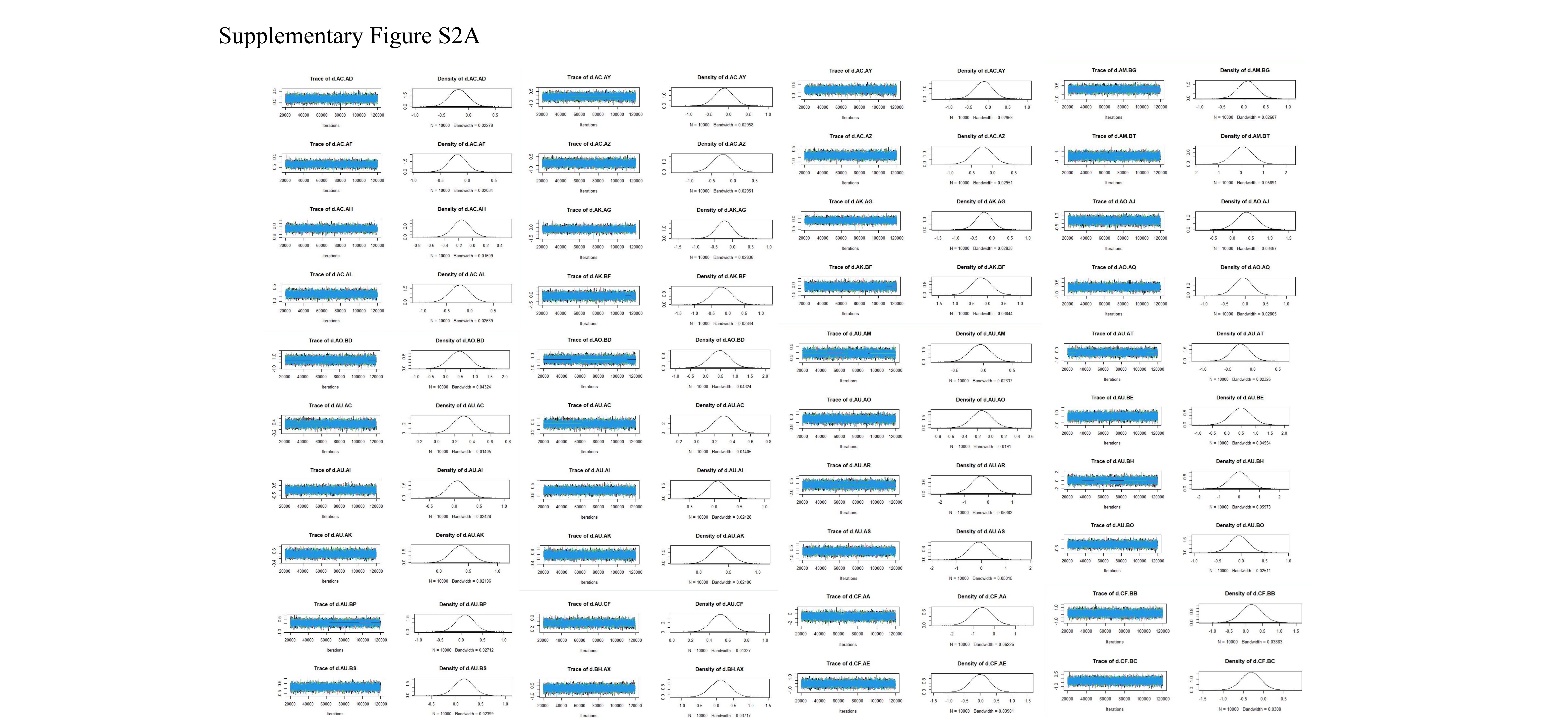

Supplement: Supplementary file 1 [file DataSheet1.zip › Supplementary figures/Supplementary figures_02A.jpg]

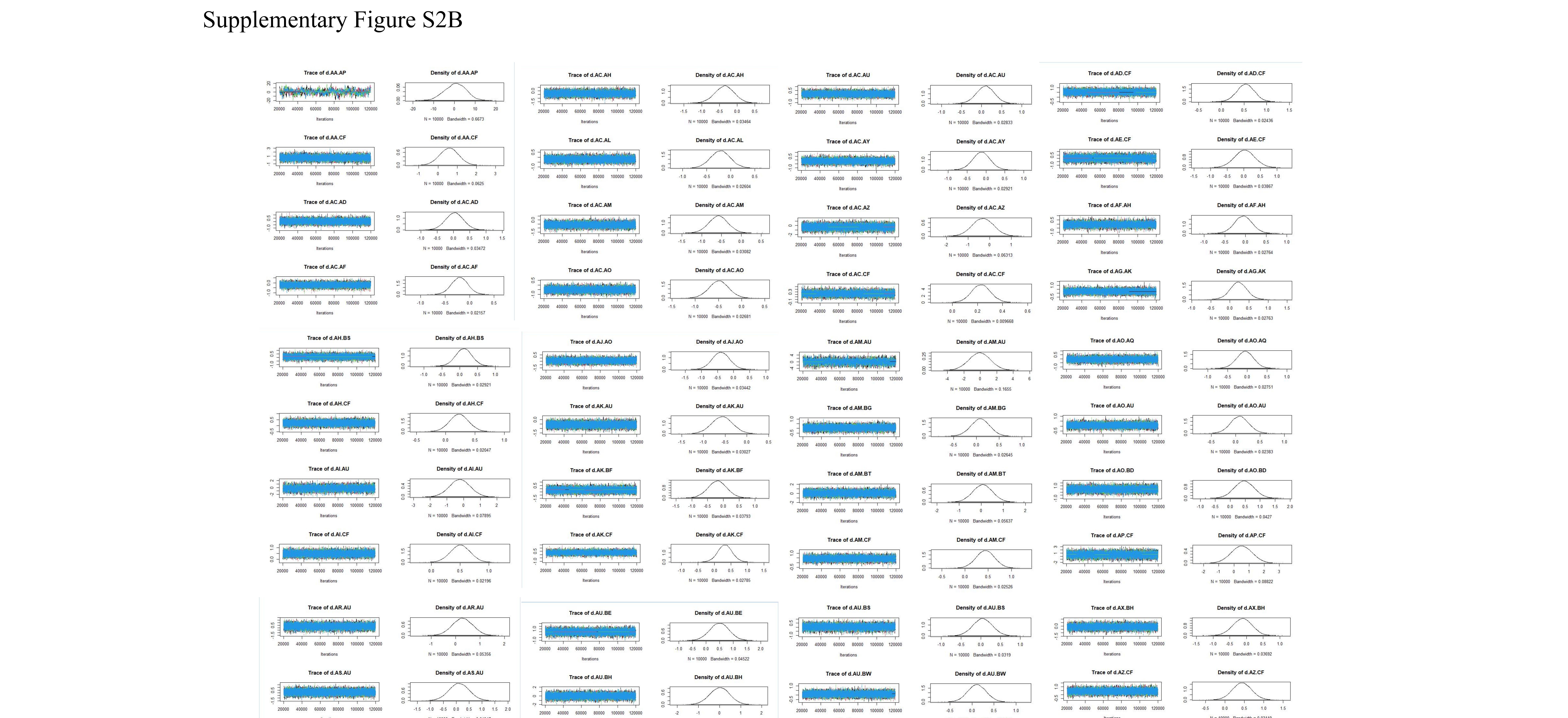

Supplement: Supplementary file 1 [file DataSheet1.zip › Supplementary figures/Supplementary figures_02B.jpg]

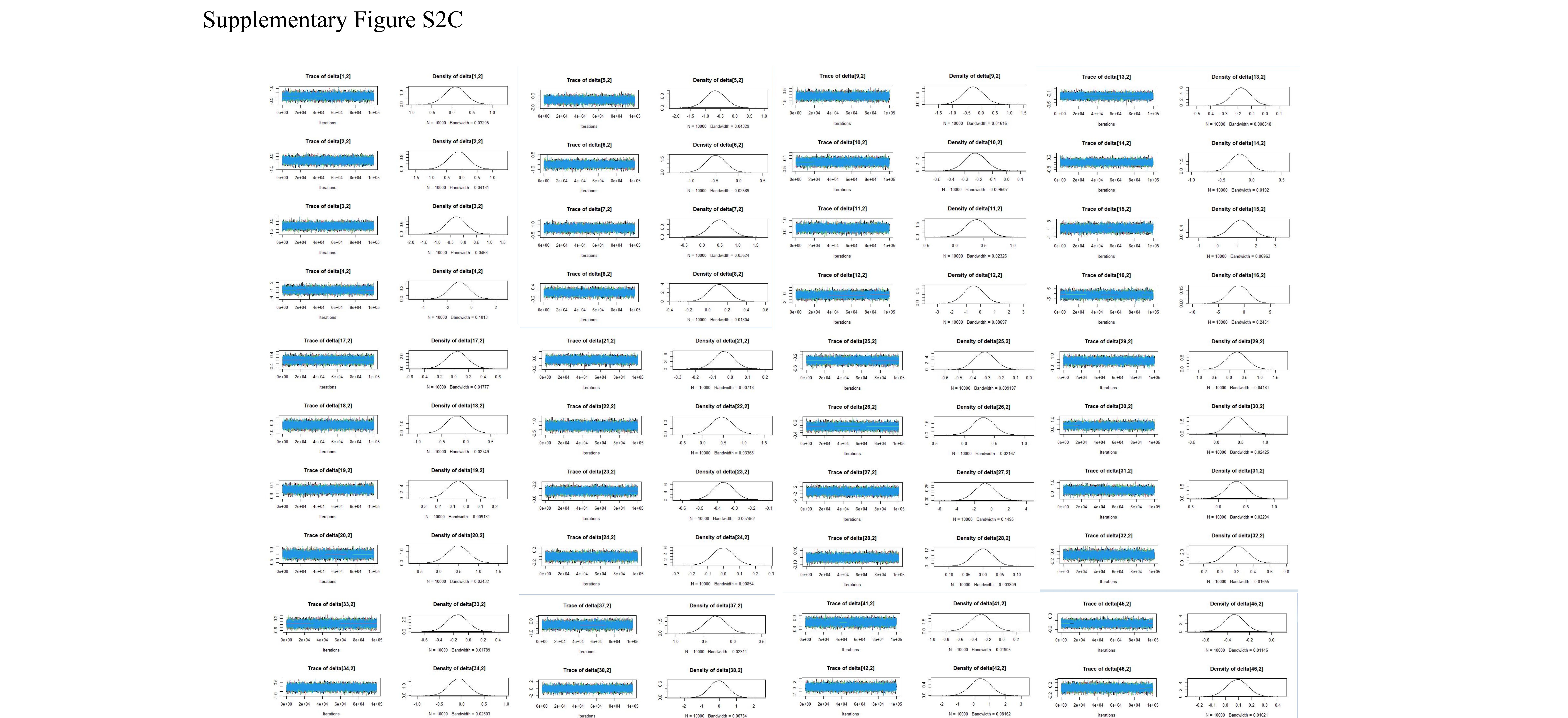

Supplement: Supplementary file 1 [file DataSheet1.zip › Supplementary figures/Supplementary figures_02C.jpg]

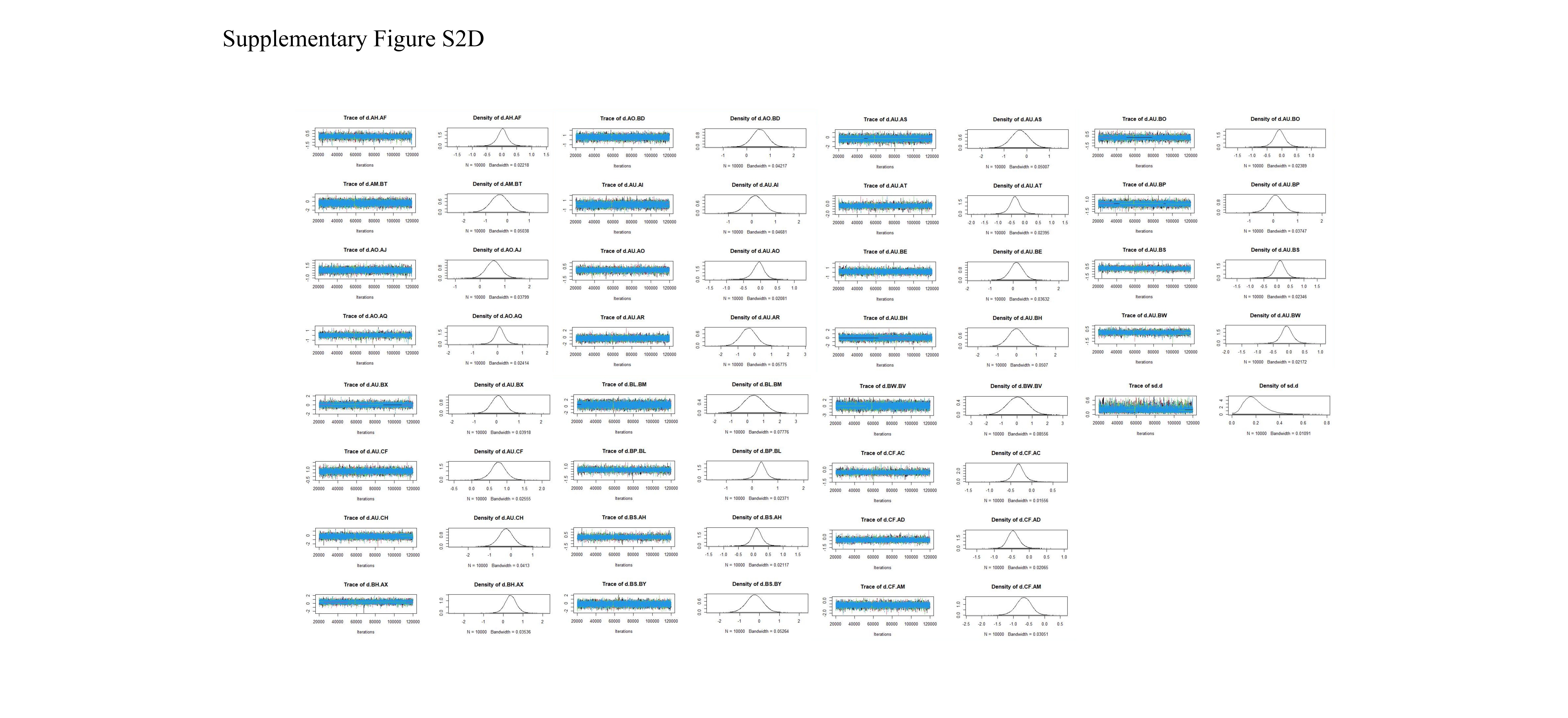

Supplement: Supplementary file 1 [file DataSheet1.zip › Supplementary figures/Supplementary figures_02D.jpg]

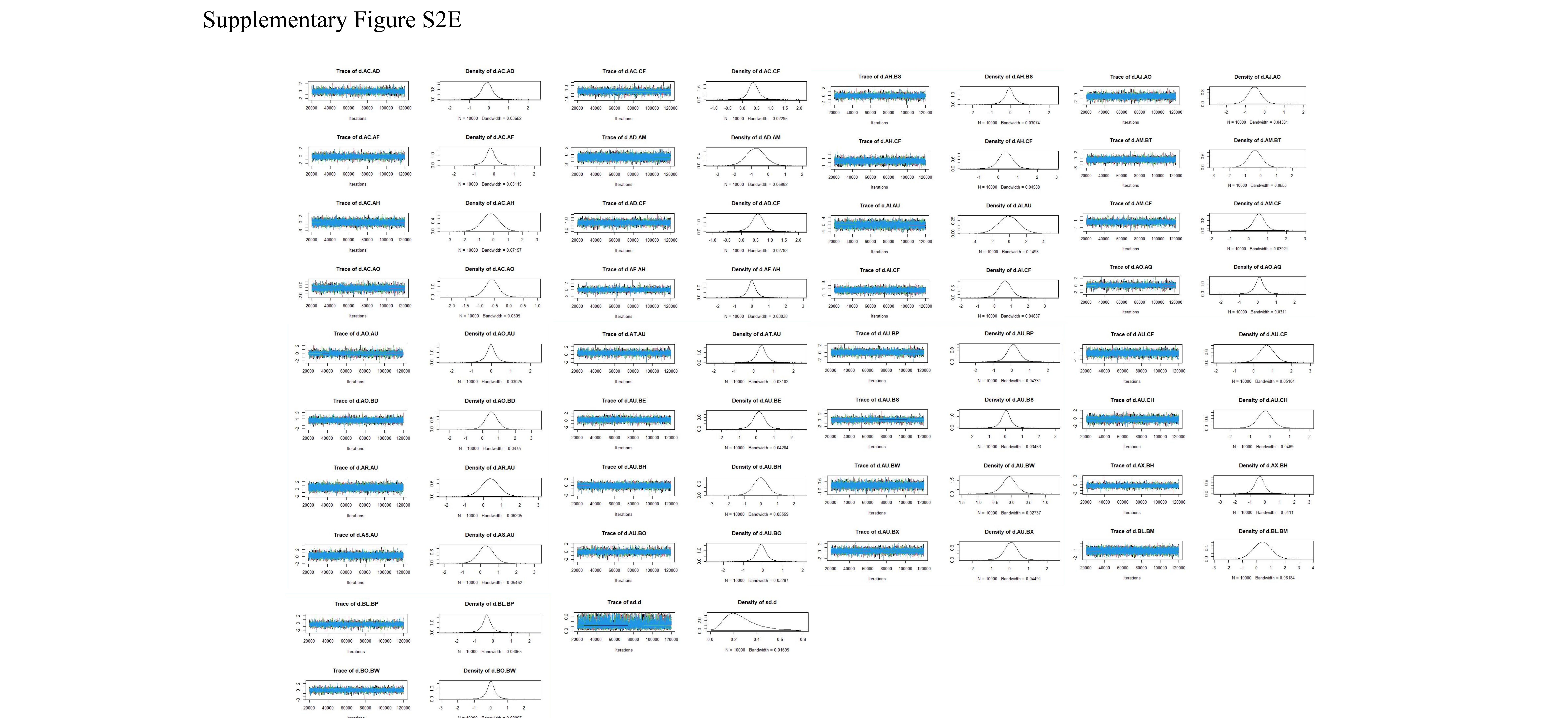

Supplement: Supplementary file 1 [file DataSheet1.zip › Supplementary figures/Supplementary figures_02E.jpg]

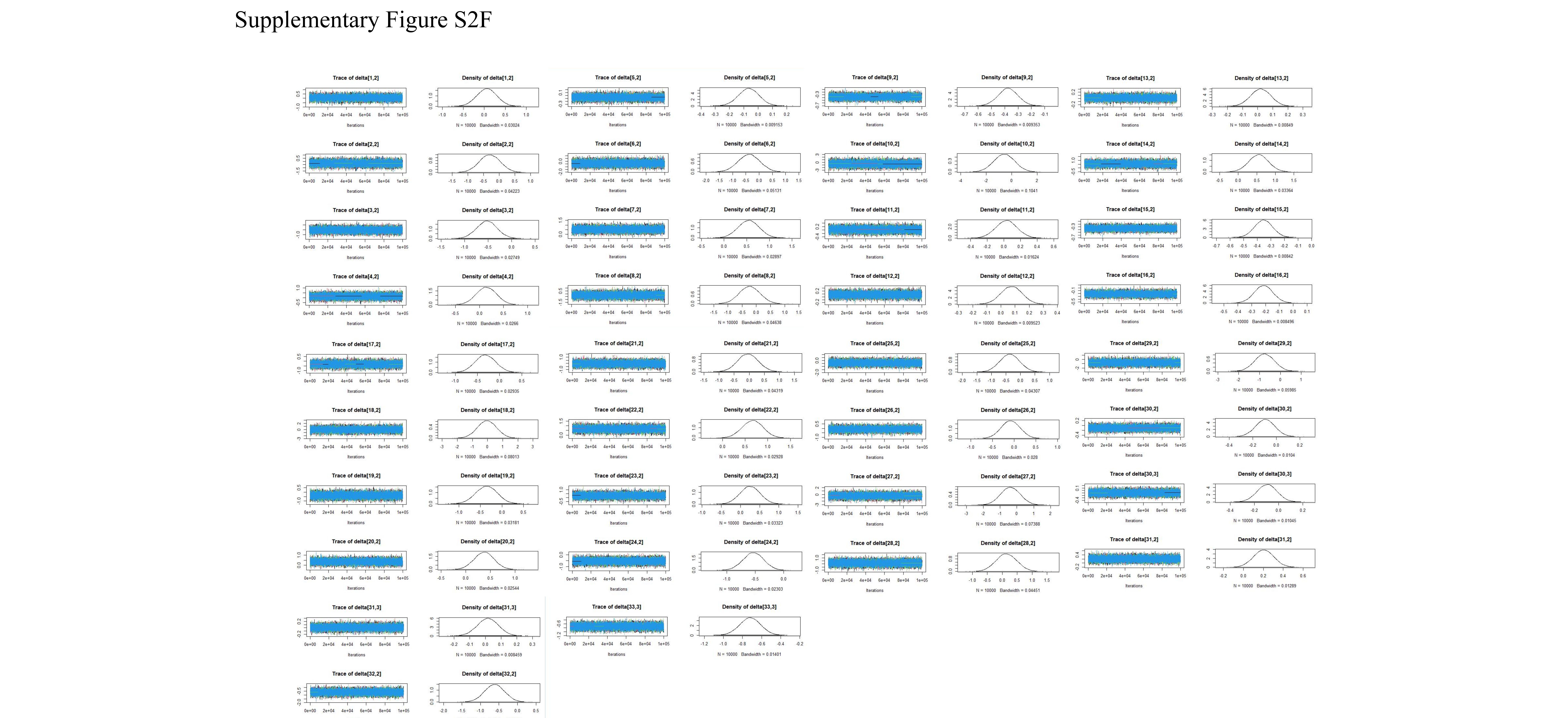

Supplement: Supplementary file 1 [file DataSheet1.zip › Supplementary figures/Supplementary figures_02F.jpg]

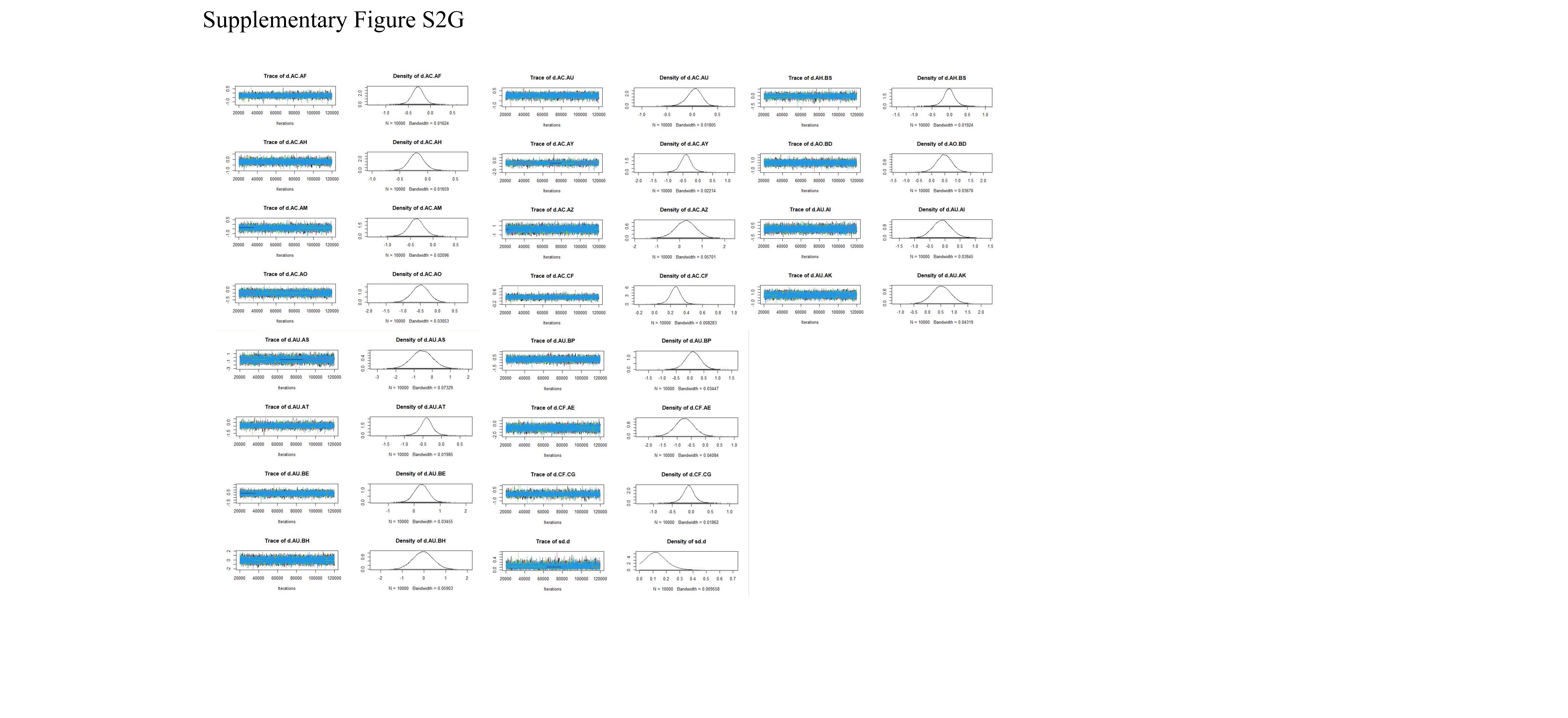

Supplement: Supplementary file 1 [file DataSheet1.zip › Supplementary figures/Supplementary figures_02G.jpg]

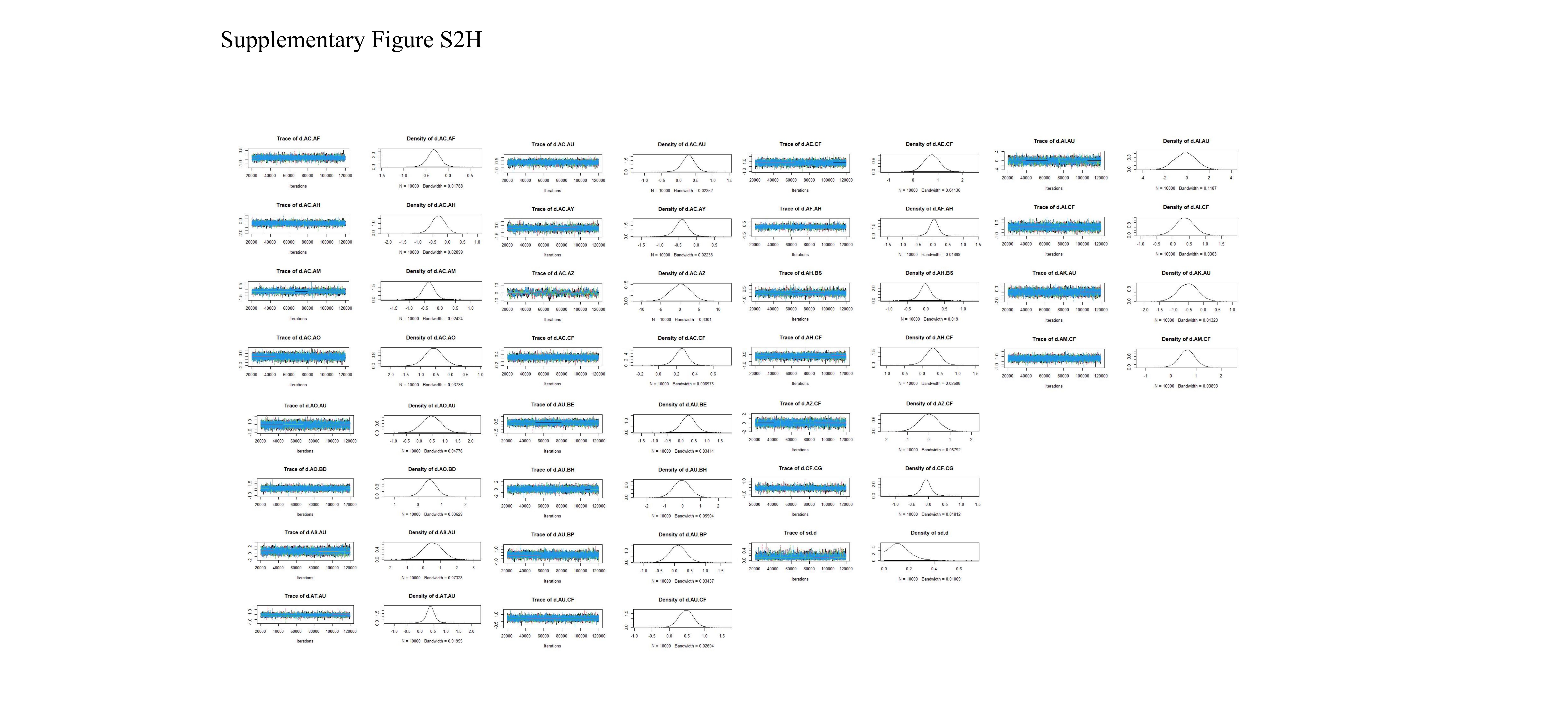

Supplement: Supplementary file 1 [file DataSheet1.zip › Supplementary figures/Supplementary figures_02H.jpg]

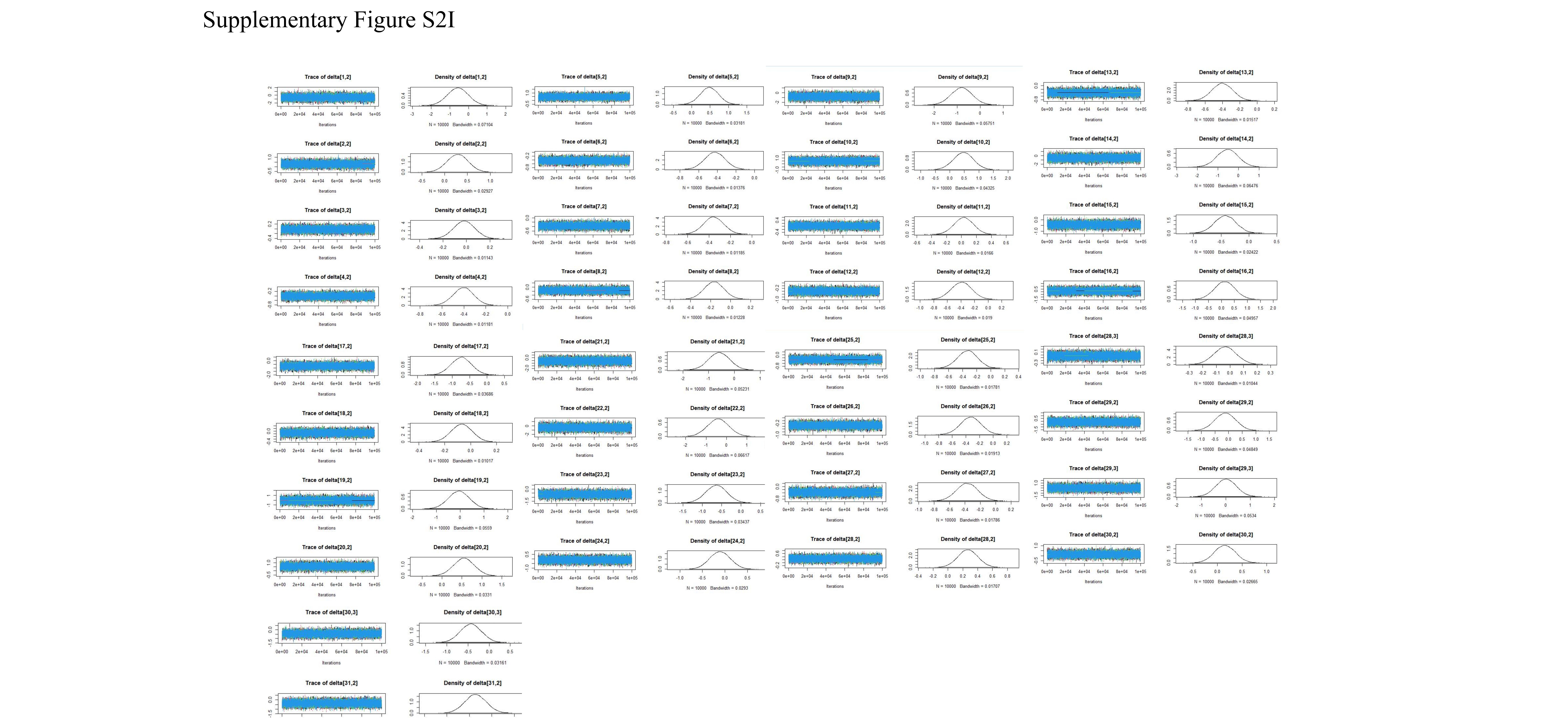

Supplement: Supplementary file 1 [file DataSheet1.zip › Supplementary figures/Supplementary figures_02I.jpg]

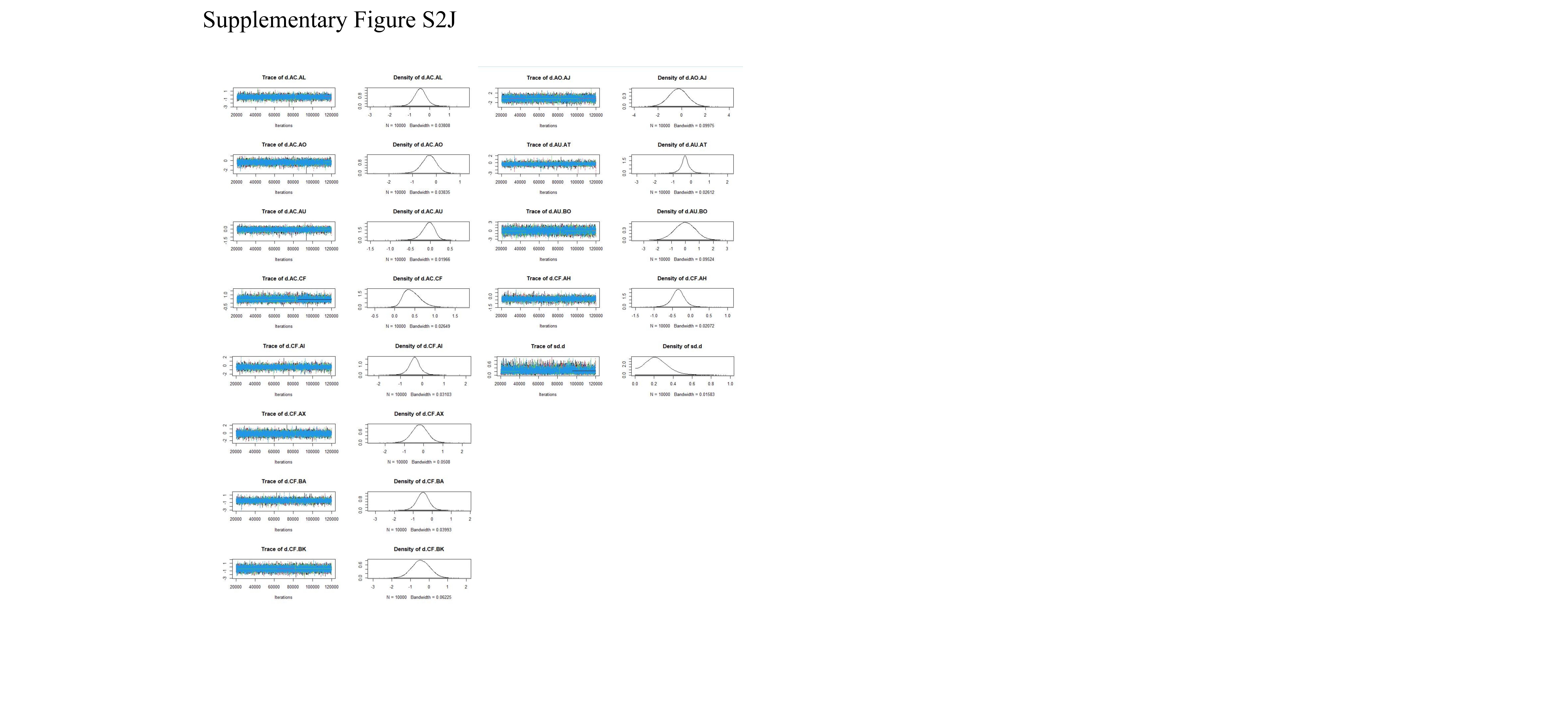

Supplement: Supplementary file 1 [file DataSheet1.zip › Supplementary figures/Supplementary figures_02J.jpg]

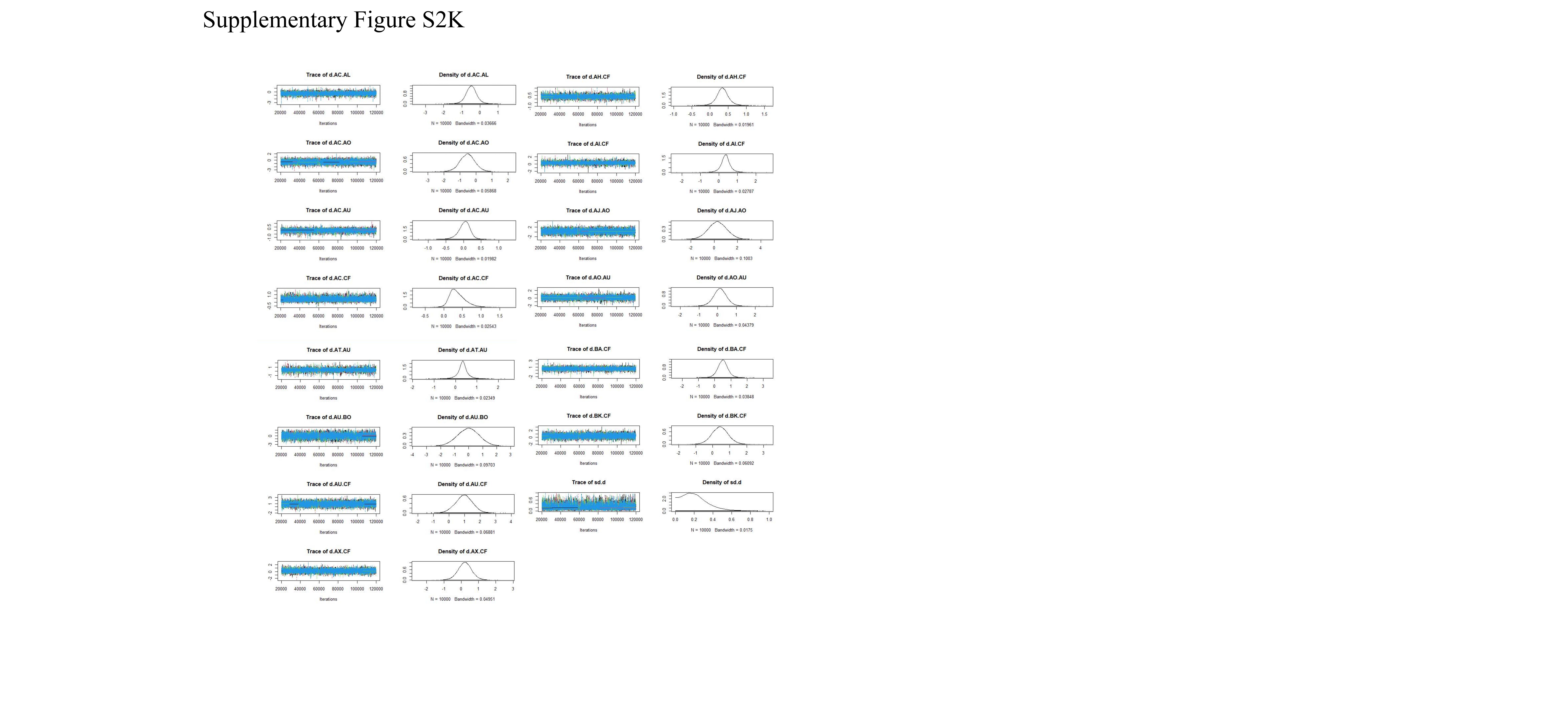

Supplement: Supplementary file 1 [file DataSheet1.zip › Supplementary figures/Supplementary figures_02K.jpg]

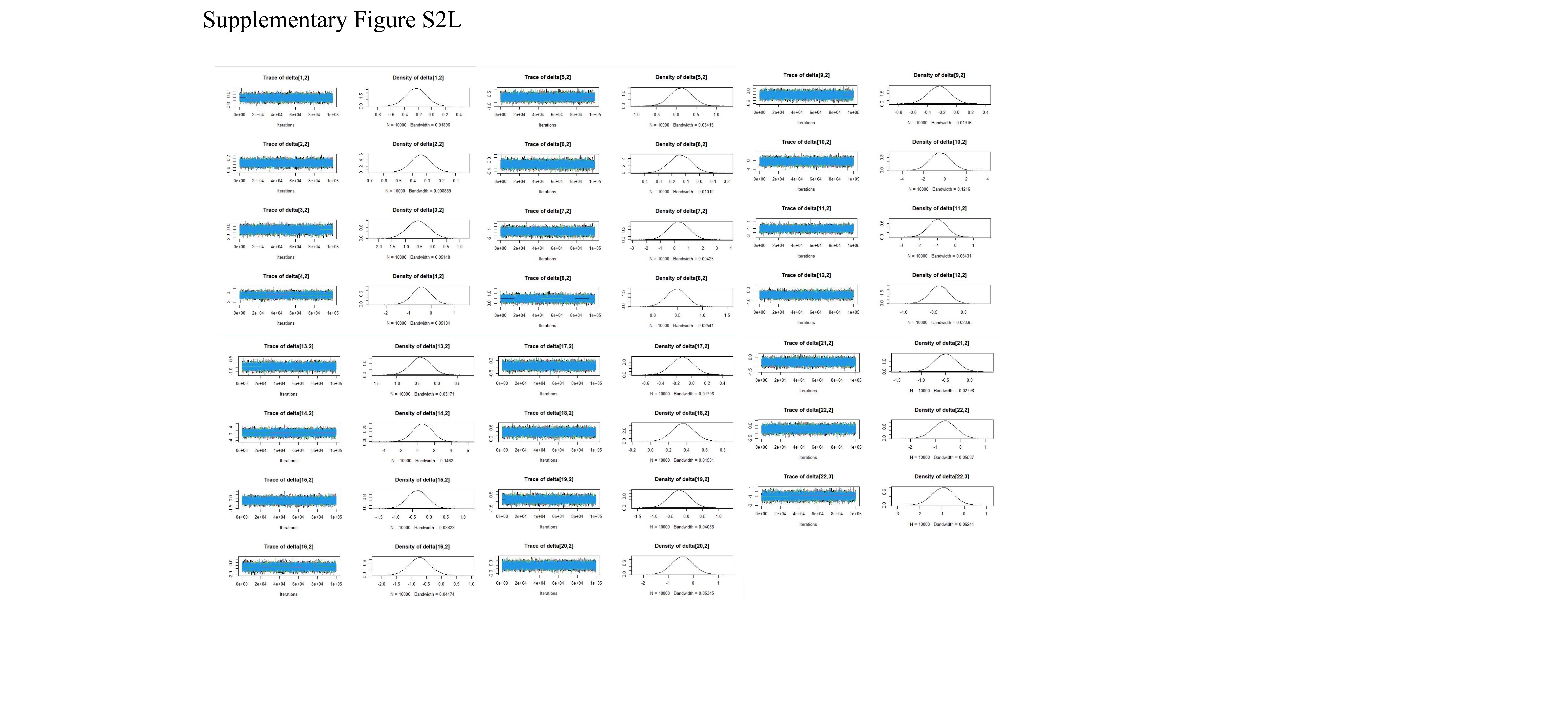

Supplement: Supplementary file 1 [file DataSheet1.zip › Supplementary figures/Supplementary figures_02L.jpg]

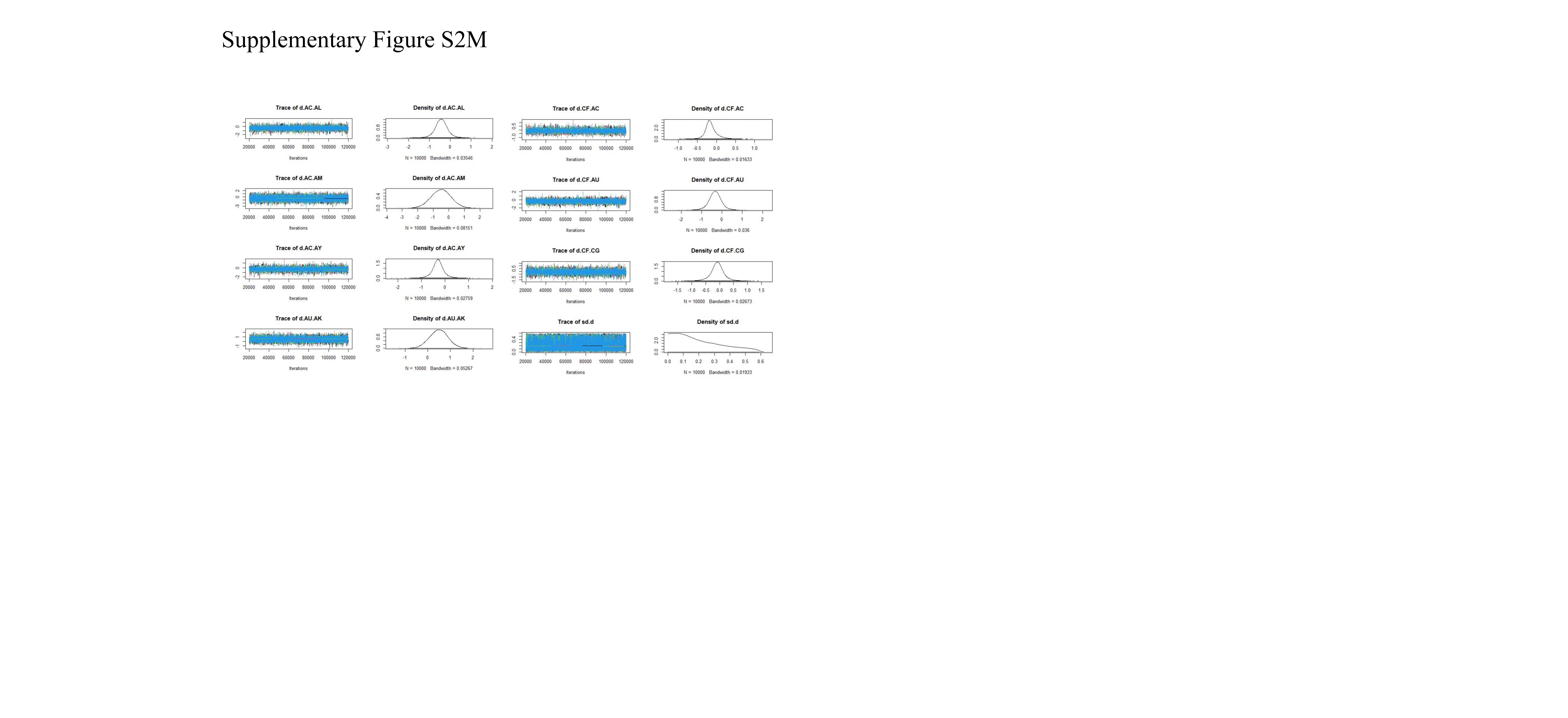

Supplement: Supplementary file 1 [file DataSheet1.zip › Supplementary figures/Supplementary figures_02M.jpg]

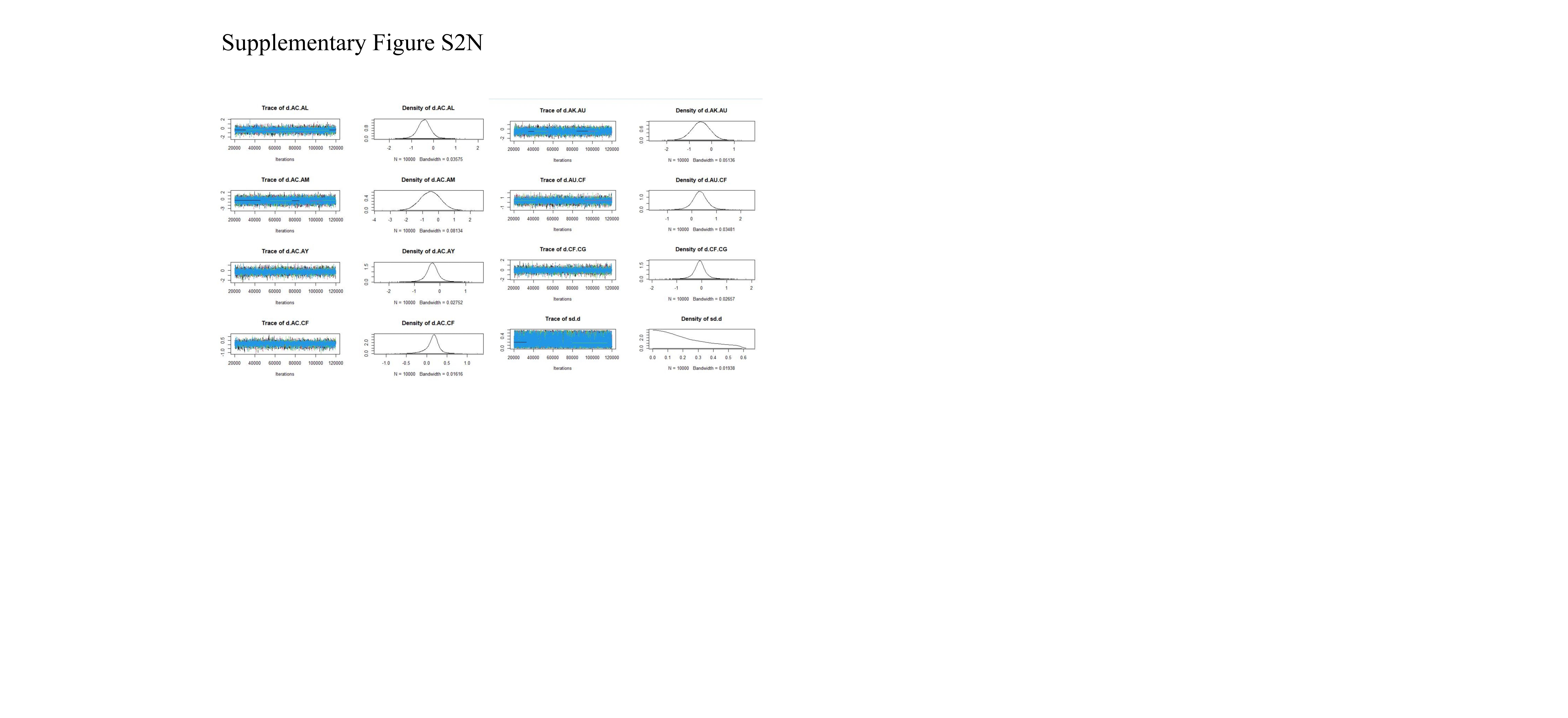

Supplement: Supplementary file 1 [file DataSheet1.zip › Supplementary figures/Supplementary figures_02N.jpg]

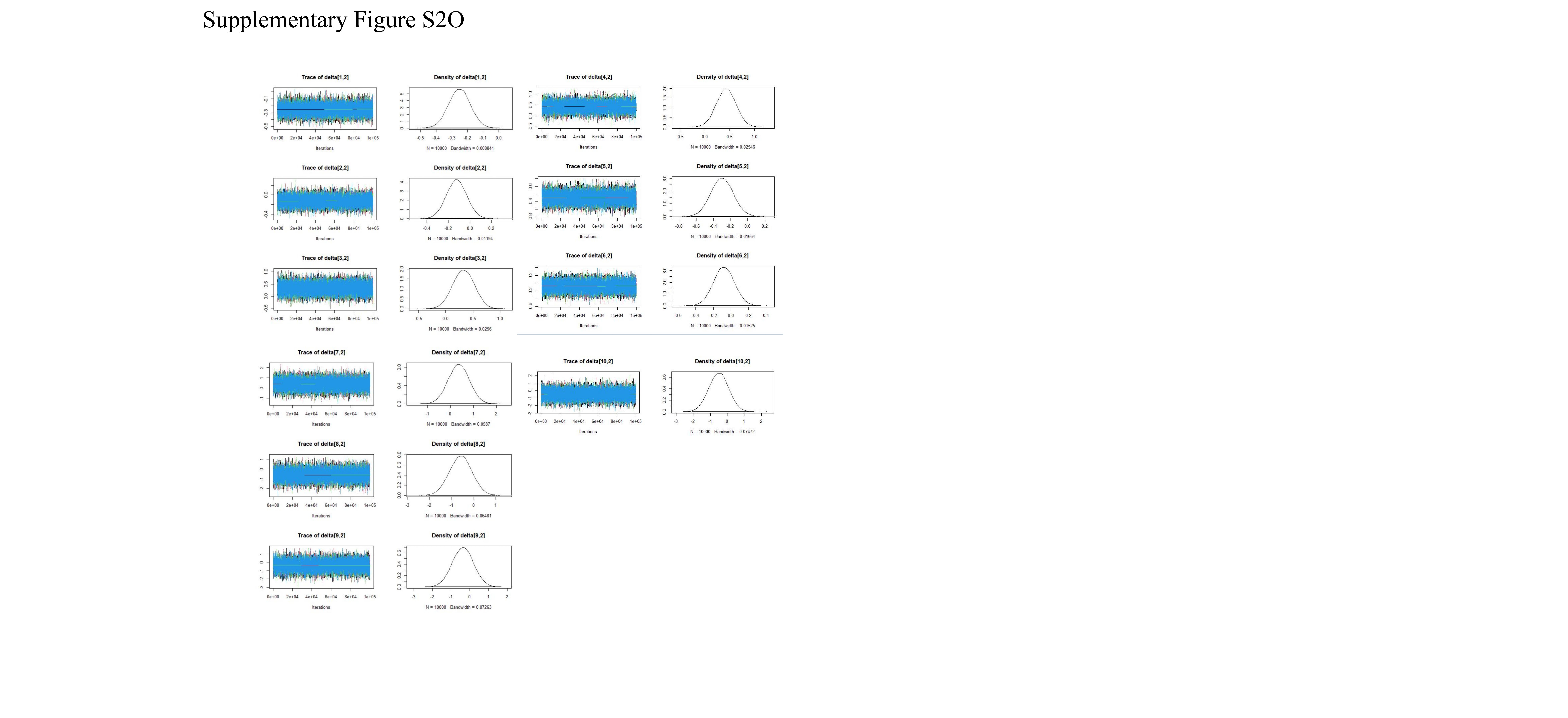

Supplement: Supplementary file 1 [file DataSheet1.zip › Supplementary figures/Supplementary figures_02O.jpg]

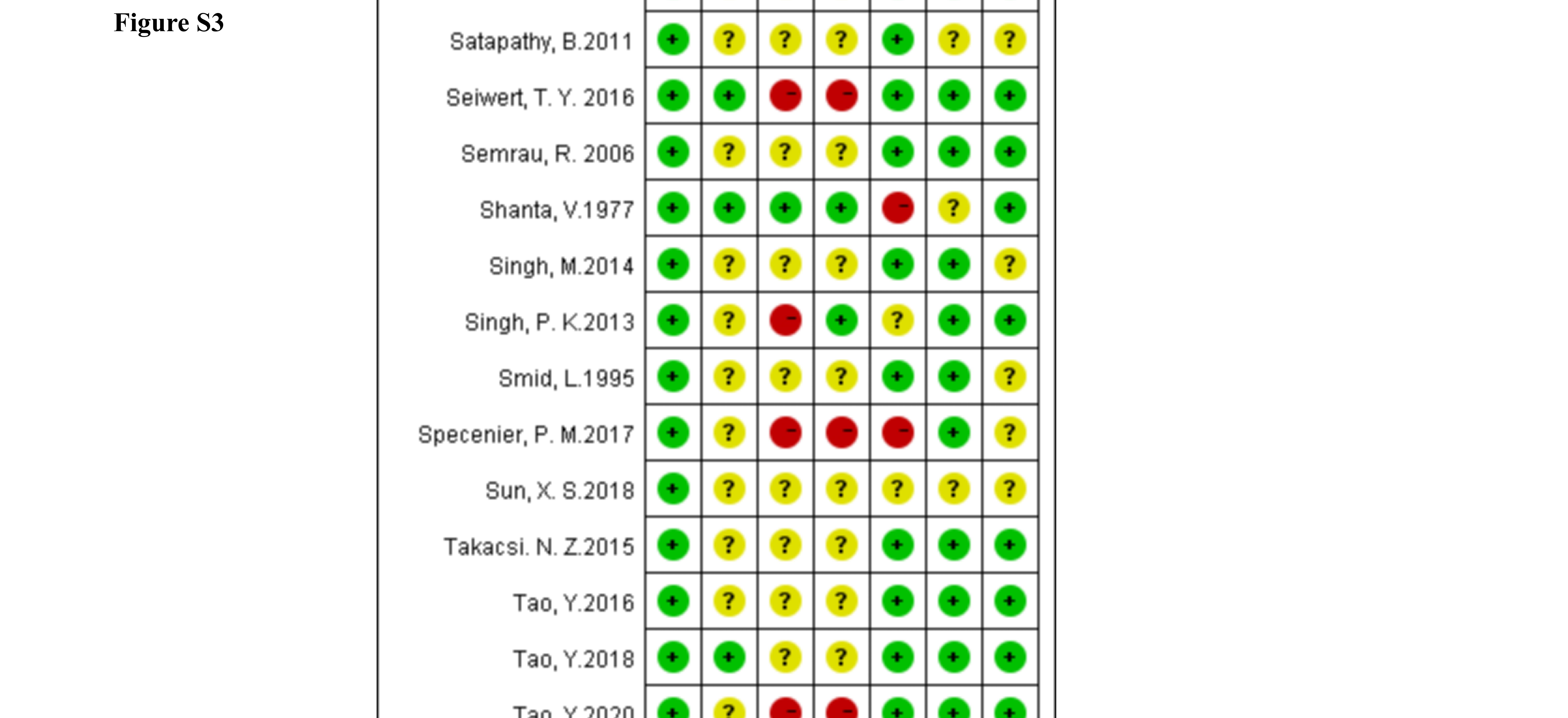

Supplement: Supplementary file 1 [file DataSheet1.zip › Supplementary figures/Supplementary figures_03.jpg]

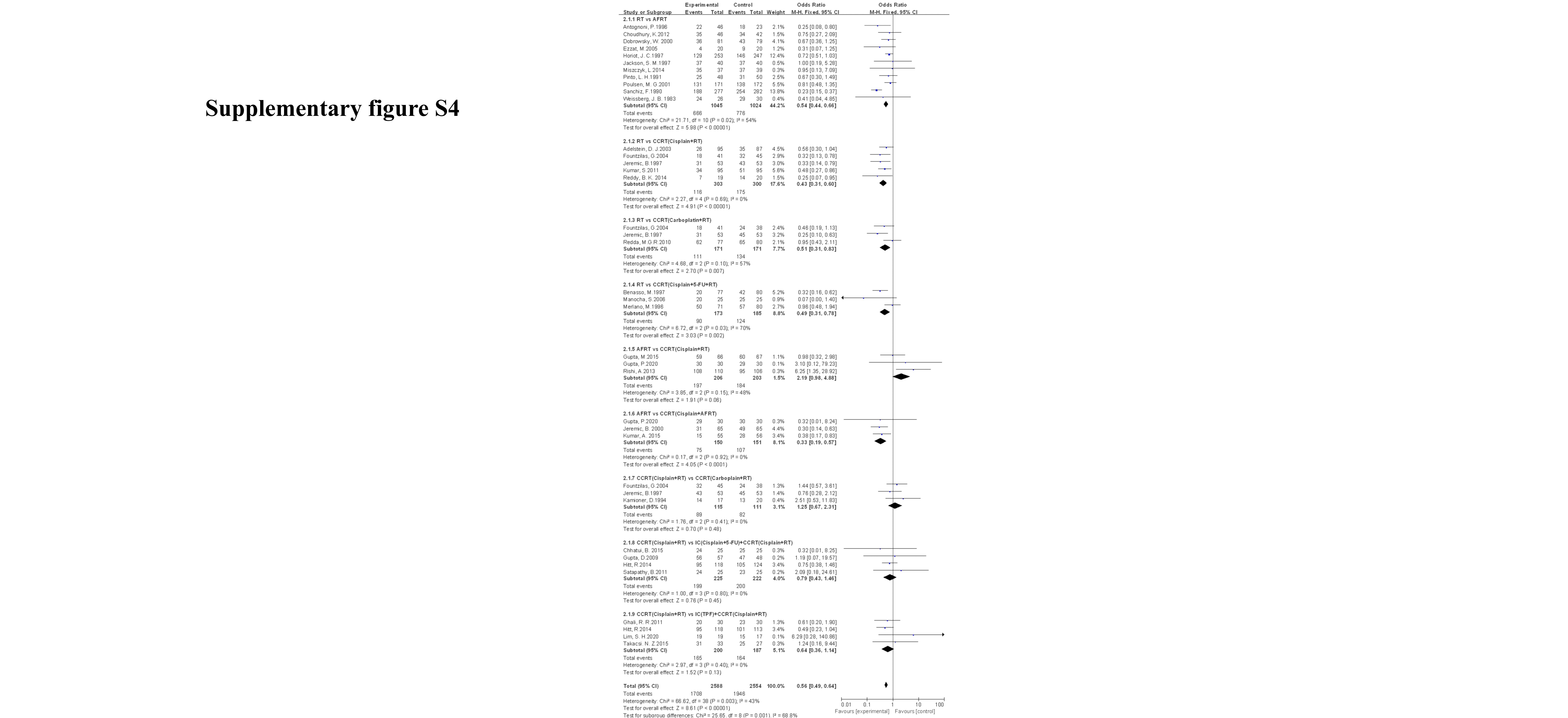

Supplement: Supplementary file 1 [file DataSheet1.zip › Supplementary figures/Supplementary figures_04.jpg]

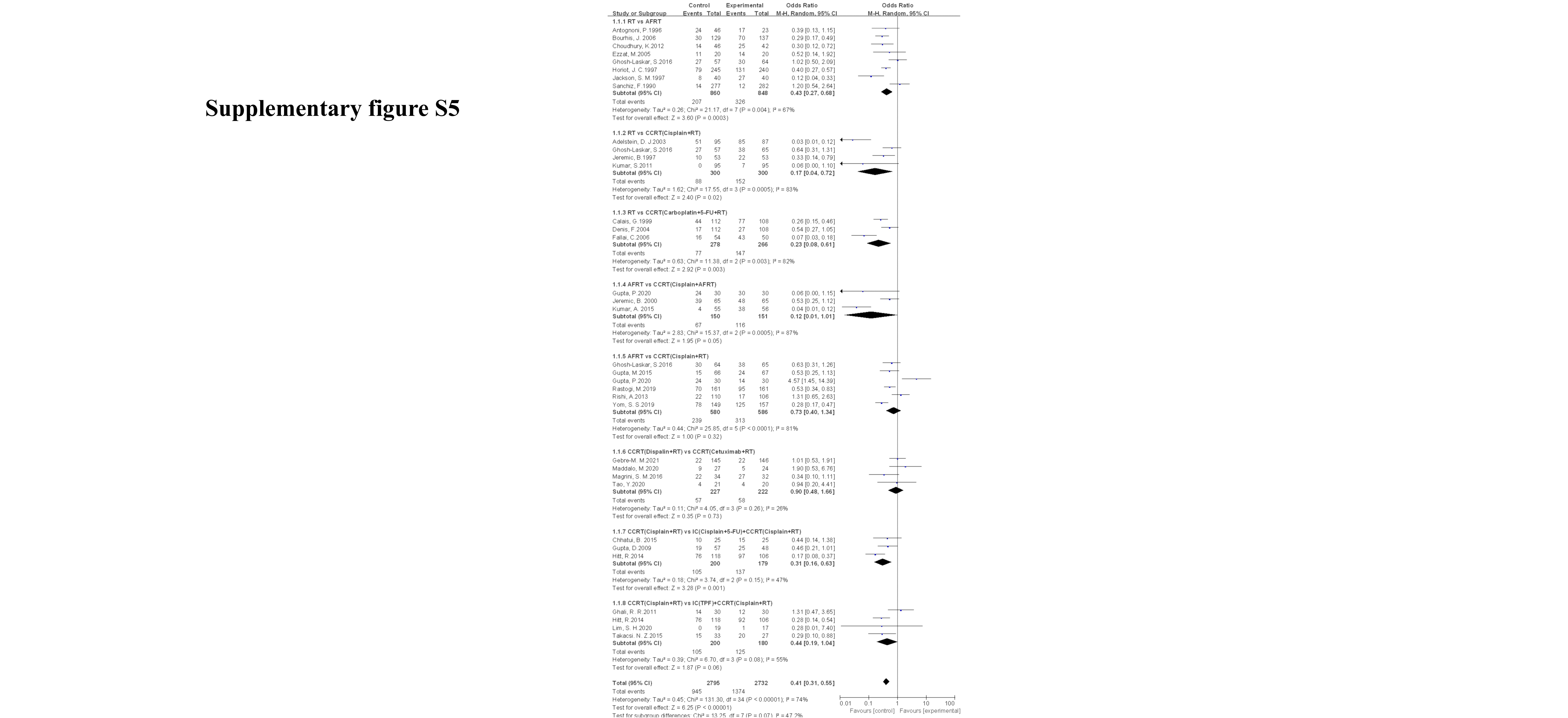

Supplement: Supplementary file 1 [file DataSheet1.zip › Supplementary figures/Supplementary figures_05.jpg]

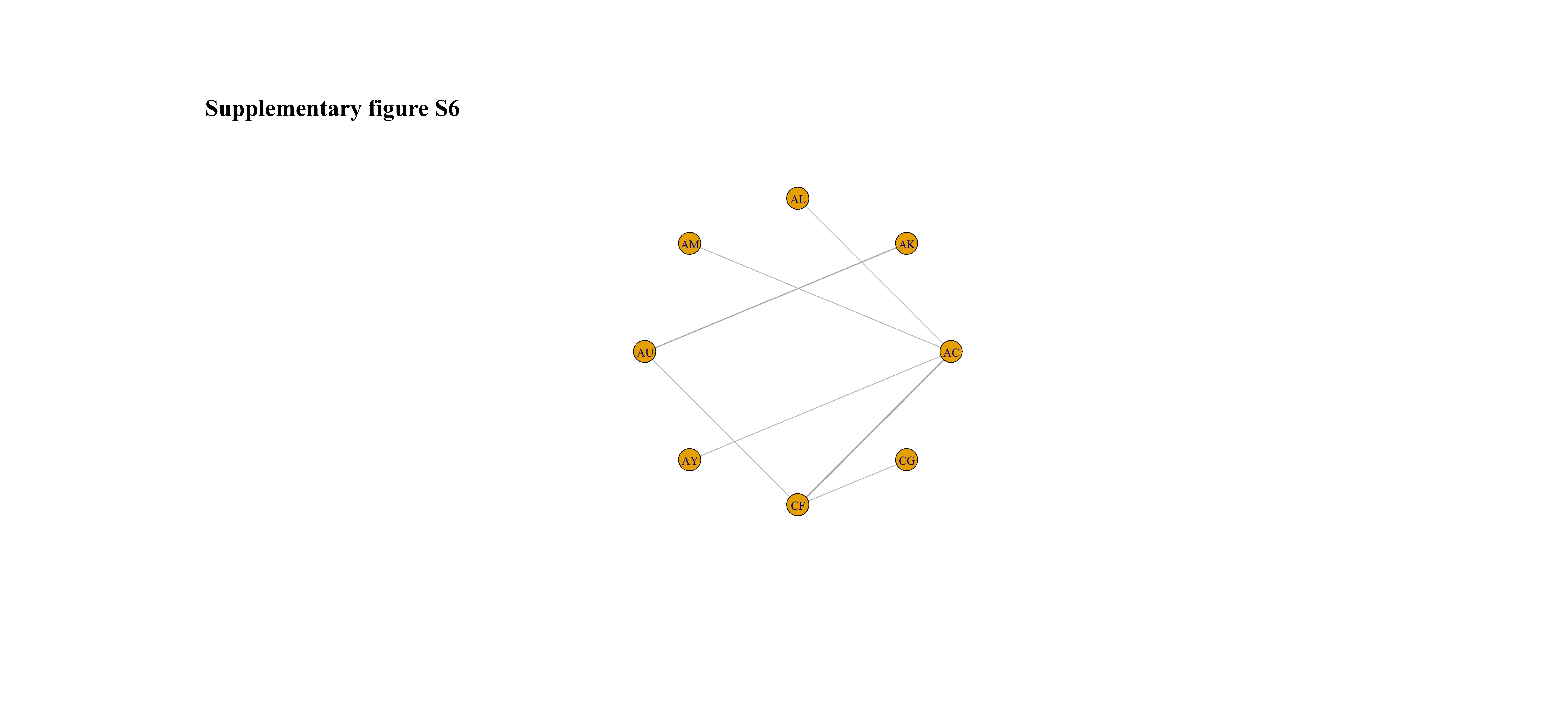

Supplement: Supplementary file 1 [file DataSheet1.zip › Supplementary figures/Supplementary figures_06.jpg]

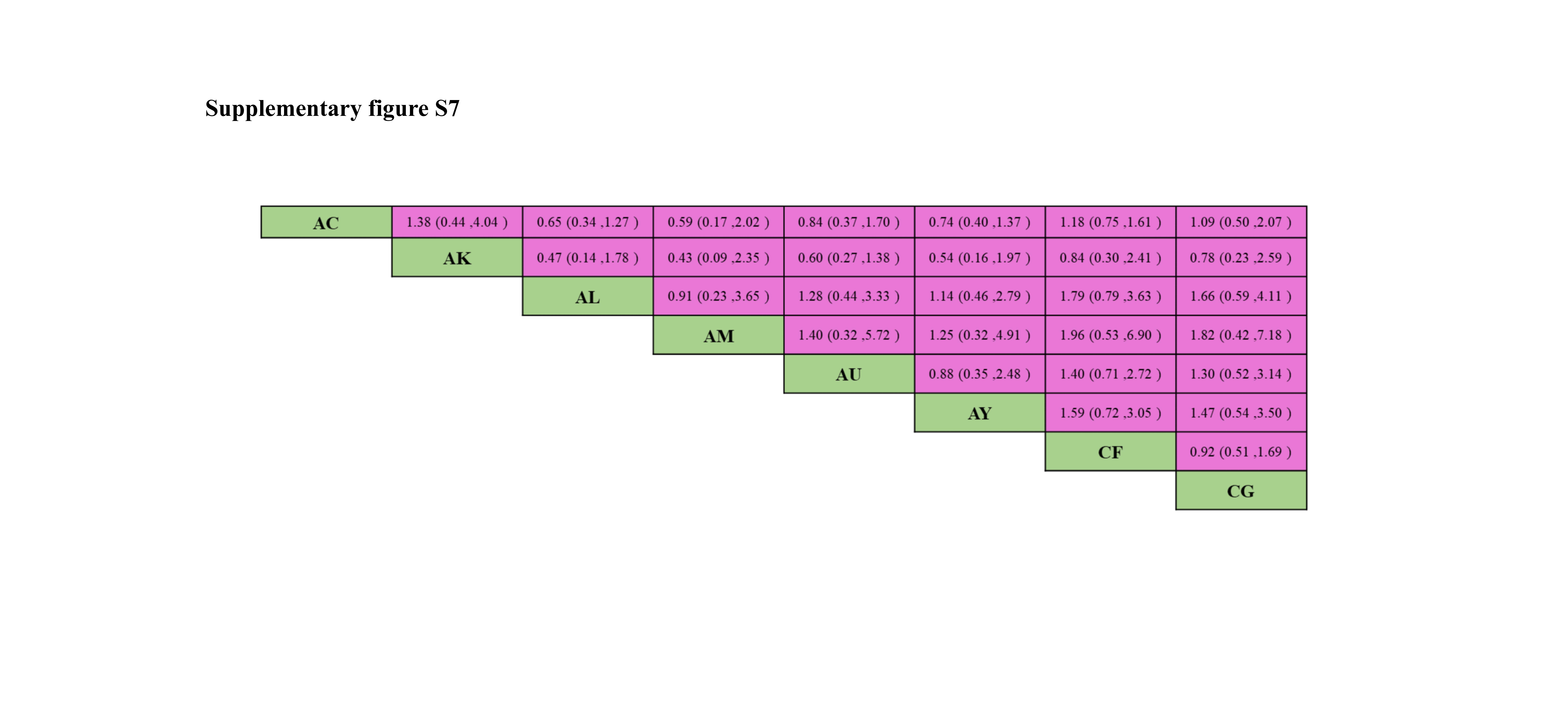

Supplement: Supplementary file 1 [file DataSheet1.zip › Supplementary figures/Supplementary figures_07.jpg]

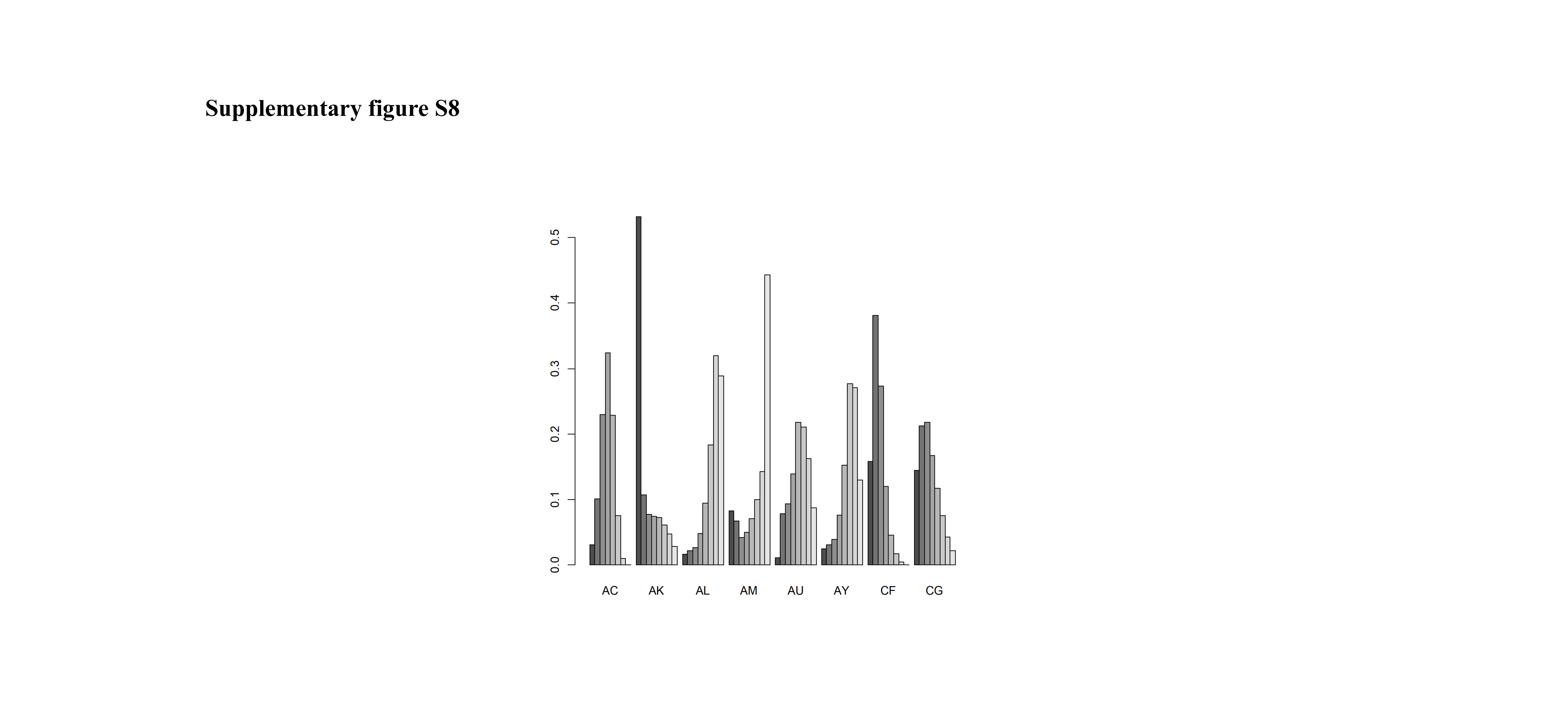

Supplement: Supplementary file 1 [file DataSheet1.zip › Supplementary figures/Supplementary figures_08.jpg]

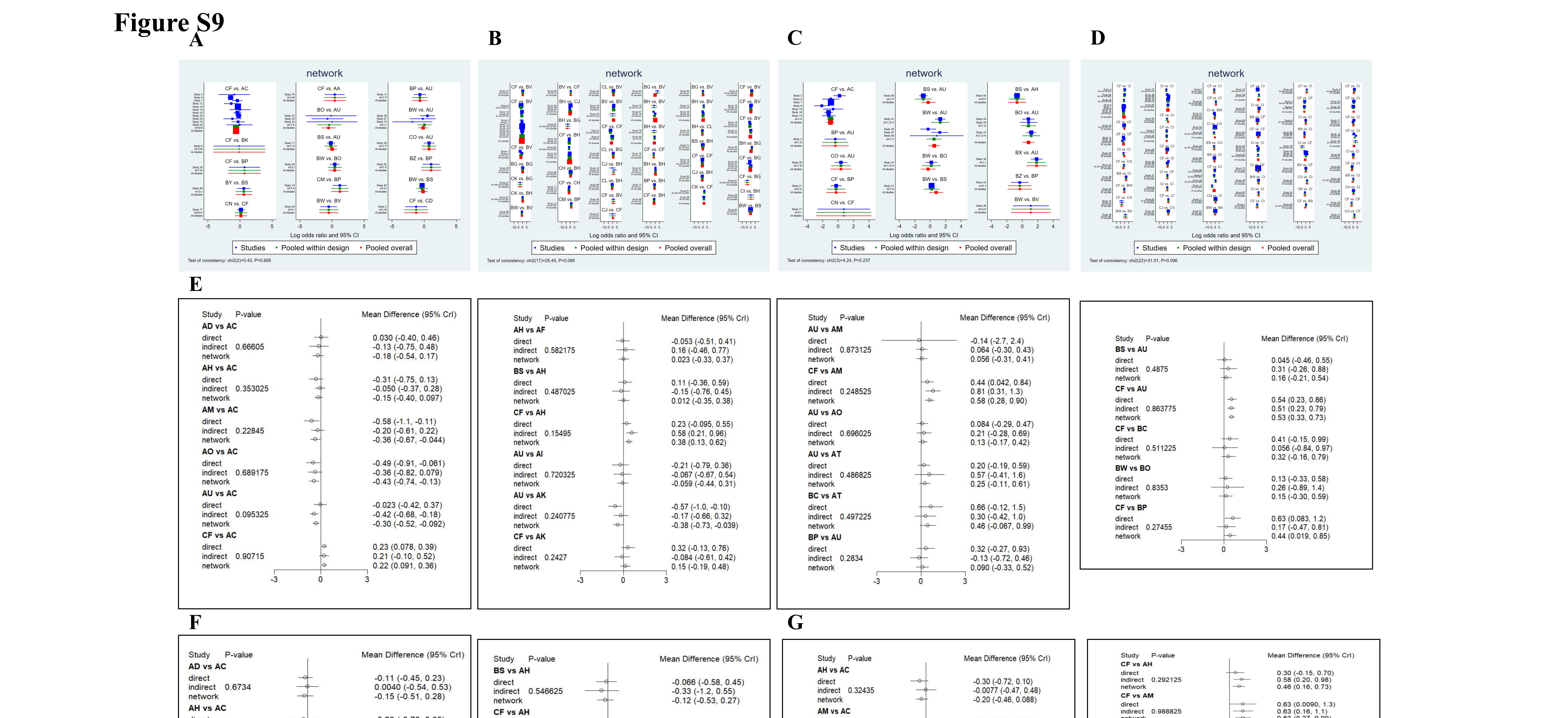

Supplement: Supplementary file 1 [file DataSheet1.zip › Supplementary figures/Supplementary figures_09.jpg]

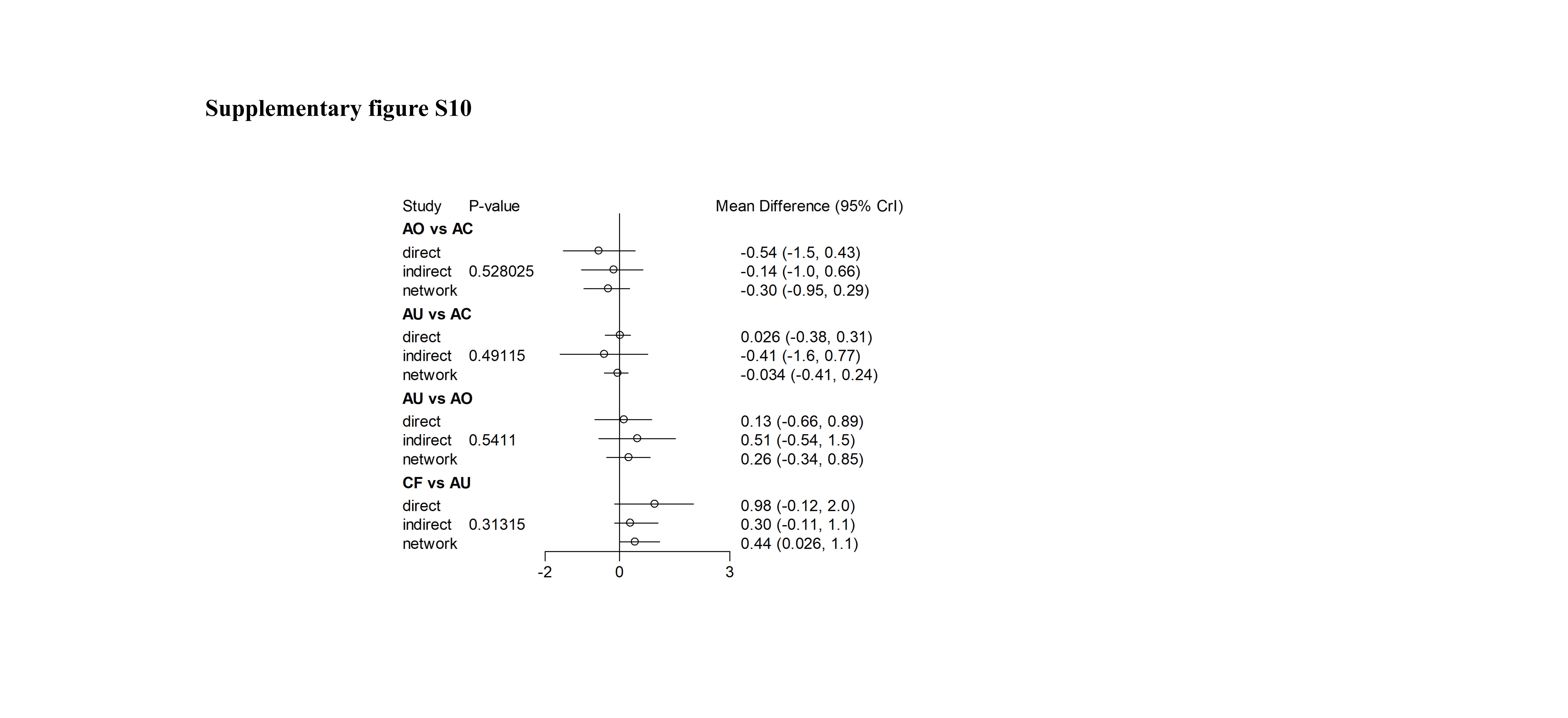

Supplement: Supplementary file 1 [file DataSheet1.zip › Supplementary figures/Supplementary figures_10.jpg]

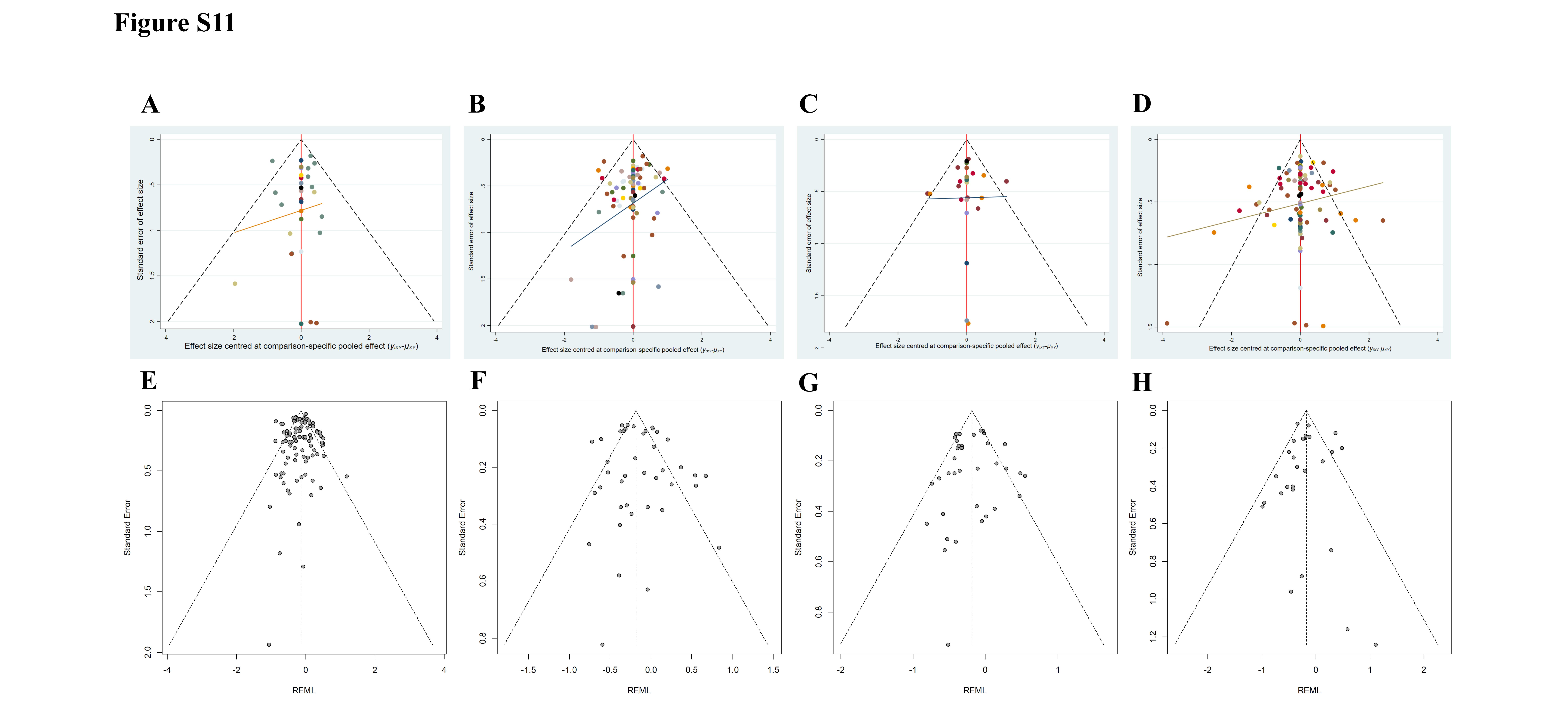

Supplement: Supplementary file 1 [file DataSheet1.zip › Supplementary figures/Supplementary figures_11.jpg]

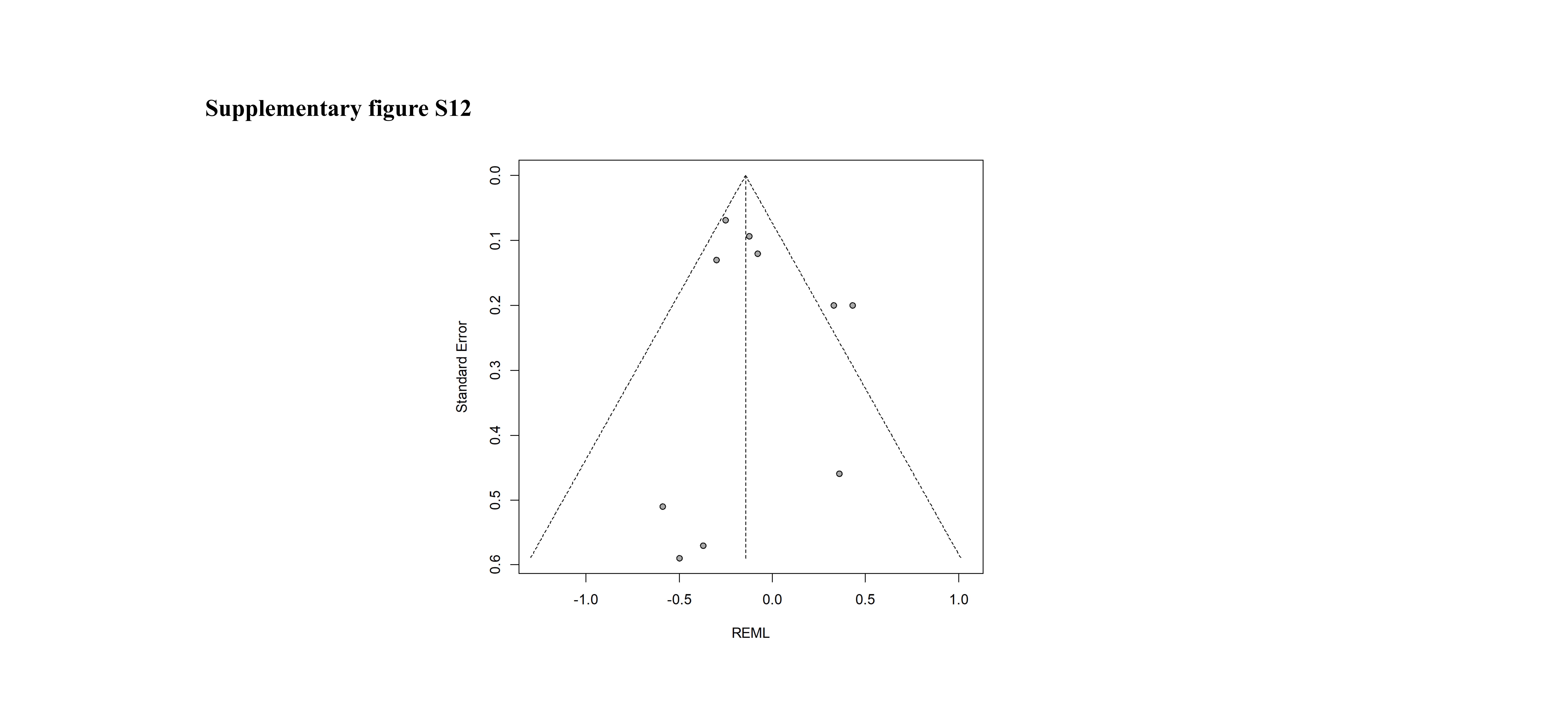

Supplement: Supplementary file 1 [file DataSheet1.zip › Supplementary figures/Supplementary figures_12.jpg]
